# Plac1 Remodels the Tumor Immune Evasion Microenvironment and Predicts Therapeutic Response in Head and Neck Squamous Cell Carcinoma

**DOI:** 10.3389/fonc.2022.919436

**Published:** 2022-06-24

**Authors:** Xiaoyan Meng, Zhonglong Liu, Lingfang Zhang, Yue He

**Affiliations:** ^1^Department of Oral Maxllofacial & Head and Neck Oncology, Shanghai Ninth People’s Hospital, Shanghai Jiao Tong University School of Medicine, College of Stomatology, Shanghai, China; ^2^National Center for Stomatology, Shanghai, China; ^3^National Clinical Research Center for Oral Diseases, Shanghai, China; ^4^Shanghai Key Laboratory of Stomatology, Shanghai, China; ^5^R&D Department, Suzhou Lingdian Biotechnology Co., Ltd., Suzhou, China

**Keywords:** head and neck cancer, tumor microenvironment, immunotherapy, Plac1, cancer testis antigen

## Abstract

Head and neck squamous cell carcinoma (HNSCC or HNSC) is the sixth most common cancer worldwide. Placenta-specific 1 (Plac1) belongs to the cancer testis antigen family and is highly expressed in malignant cells in HNSC. However, the biological function and prognostic value of plac1 in HNSC are still unclear. In the current research, we performed a comprehensive analysis of plac1 using The Cancer Genome Atlas (TCGA) and Gene Expression Omnibus (GEO) bulk RNA sequencing databases as well as a single-cell sequencing dataset. We constructed a 15-gene prognostic signature through screening plac1-related immunomodulators and validated its efficiency and accuracy in immunotherapy cohorts and a pancancer database. We found that plac1 expression level is a prognostic predictor of poor overall survival in patients with HNSC. Plac1 is associated with epithelial–mesenchymal transition and tumor invasion. Plac1 has a “dual immunosuppressive function” on tumor microenvironment. On one hand, plac1-positive cells promote extracellular matrix formation and suppress immune cell infiltration. On the other hand, plac1-positive cells enhance the interaction between dendritic cells and macrophages, which further suppresses antitumor immunity. Finally, we constructed a 15-gene prognostic signature, the efficiency and accuracy of which were validated in immunotherapy cohorts and a pancancer database. In conclusion, plac1 is a promising candidate biomarker for prognosis, a potential target for immunotherapy, and a novel point for studying the immunosuppressive mechanisms of the tumor microenvironment in HNSC.

## Introduction

Head and neck squamous cell carcinoma (HNSCC or HNSC) is the sixth most common cancer worldwide and includes cancer in the oral cavity, oropharynx, nasopharynx, hypopharynx, and larynx (1). The mainstream therapy is surgical resection, and chemotherapy and radiotherapy can be used as adjuvant therapy for locally advanced or recurrent/metastatic cancer. In recent years, immunotherapy, such as cancer vaccines, adoptive cell therapy, and immune checkpoint blockers (ICBs), has emerged as a novel cancer therapy and has shown promising results in multiple clinical trials (2). However, occult mechanisms impact the objective response rate, which refers to primary and adaptive resistance and needs further elucidation (3).

Cancer/testis antigens (CTAs) are a group of genes that are restrictedly expressed in malignant cells as well as some germline cells. These expression characteristics make CTAs promising candidates for antitumor therapy targets (4, 5). Placenta-specific 1 (plac1) belongs to the CTA family. In humans, plac1 encodes a protein containing 212 amino acids and is expressed mainly in trophoblastic membrane under normal conditions. Plac1 expression has been observed in multiple cancer cells, including breast (6), lung (7), stomach (8), and colon cancers (9). Moreover, the association between plac1 expression and invasive phenotype of cancer cell was confirmed by *in vitro* experiments, but the mechanism behind has not been elucidated to date (10, 11).

In this study, we performed a comprehensive analysis of plac1 expression patterns (the workflow is shown in **Supplementary Figure S1**). We found that the plac1 mRNA expression level is a prognostic predictor of poor overall survival in patients with HNSC. Then, we utilized a single-cell sequencing method to analyze cell–cell interactions and plac1-positive cell characteristics. Regarding cancer biological functions, plac1 is associated with epithelial–mesenchymal transition, promoting cancer invasion and metastasis. Based on the analysis of bulk and single-cell sequencing data, we found that the impact of plac1 on the tumor microenvironment (TME) could be summarized as a “dual immunosuppressive function”. The immunosuppressive effects of plac1 represent characteristics of “cold tumors” related to poor outcomes of immunotherapy. We confirmed this conclusion in various immunotherapy cohorts. Finally, we screened and constructed a 15-gene prognostic signature, the efficiency and accuracy of which were validated in an immunotherapy cohort as well as a pancancer database.

## Materials and Methods

### Immunohistochemistry Staining of HNSC Microarray

The tissue microarray chips were obtained from Xi’an Bioaitech Company, and the chip comprised 45 HNSC tissue dots, three marginal lingual tissue, and six normal epithelia tissues, 53 of which could be utilized for further analysis.

The tissue microarray chips through deparaffinization and dehydration were incubated with polyclonal rabbit anti-human plac1 (dilution 1:100, Novus, NBP2-32379) overnight at 4°C after epitope retrieval, H_2_O_2_ treatment, and non-specific antigen blocking. The chips were next incubated with secondary antibody, followed by signal detection with DAB staining kit (Vector Laboratories, USA), and immunohistochemistry (IHC) score [IHC score = ∑ (PI × I) = (percentage of cells of weak intensity × 1) + (percentage of cells of moderate intensity × 2) +( percentage of cells of strong intensity × 3)] was obtained with Quant Center Analysis tool.

### Cell Culture and Transient Transfection

A total of two cell lines including human HNSC HN6 and SCC9 (American Type Culture Collection) were used in current research. They were cultured in Dulbecco’s modified Eagle’s medium (DMEM; Gibco, Carlsbad, CA, USA) containing 10% fetal bovine serum (Gibco, Carlsbad, CA, USA). The culture was maintained in a humidified incubator with 37°C, 5% CO_2_. Lipofectamine 3000 (Invitrogen, Carlsbad, CA, USA) was used to transfect the negative control and plac1 siRNAs (GenePharma, Shanghai, China) into HNSC cells according to the manufacturer’s instruction. The sequences for plac1 siRNAs were sense (5′–3′) GCUCCAUAGACUGGUUCAUTT and antisense (5′–3′) AUGAACCAGUCUAUGGAGCTT.

### Wound Healing and Transwell Assay

The migration and invasive abilities of HNSC cells were determined by wound healing and Transwell assays after transfection with siRNAs.

As for the wound healing assay, the adherent cells were scratched and photo-recorded. Then, the cells were returned to the cell incubator. After 24 h, the cells were washed twice, and five fields were randomly selected under ×40 microscope for photo-recording.

As for the Transwell assay (8.0 μm pores Transwell, Corning, USA), cells (1.0 × 10^5^ for migration and 2.0 × 10^5^ for invasion) were cultured in serum-free H-DMEM in the upper chambers. H-DMEM containing 10% fetal bovine serum was added to the lower chambers. After the cells were cultured for 24 h, cells that had migrated to the opposite side of the Transwell filter were fixed with 4% paraformaldehyde and stained with crystal violet staining solution (Beyotime, Shanghai, China). In the Transwell invasion assay, the top chamber was coated with Matrigel (1:10 in H-DMEM dilution, Corning, USA); the other procedure was the same as that of the Transwell migration assay. Five fields were randomly selected under ×100 microscope for photo-recording.

### Data Retrieval and Preprocessing

Publicly available HNSC and skin cutaneous melanoma (SKCM) datasets were obtained from The Cancer Genome Atlas (TCGA) and Gene Expression Omnibus (GEO) datasets (GSE103322, GSE137564, GSE39725, GSE39723, GSE85195, GSE65858, GSE78060, GSE30784, GSE126045, GSE78220, and GSE91061). The sample numbers that we used are summarized in **Supplementary Table S1**. Level 3 RNA-Seq data consisting of 501 HNSC tissues and 44 normal controls were downloaded from UCSC xena browser (https://xena.ucsc.edu/) (12). Non-primary tumors and formalin-fixed paraffin-embedded samples were filtered out; then, only one sample was kept for a patient. In total, 482 HNSC samples were finally included. Corresponding clinical characteristics, therapeutic regimen, corresponding response, follow-up, RNA-Seq, and somatic mutation data were obtained for TCGA-HNSC and TCGA-SKCM datasets. For the TCGA-SKCM cohort, we only kept the patients treated with immunotherapy. The details of the clinico-pathological characteristics are summarized in **Supplementary Table S3** and **Supplementary Table S4**.

Data on RNA-seq were fragments per kilobase per million or transcripts per million normalized and log_2_-transformed, while data from microarray were processed according to the normalization method suitable for the chip platform. Then, genes with low expression were eliminated. After a principal component analysis, any outlier samples were removed.

### Somatic Mutation Analysis

The somatic mutation datasets of patients in the plac1-positive and plac1-negative groups were retrieved from cbioportal pan-cancer atlas (https://www.cbioportal.org/). The mutation frequency and somatic alterations of common driver genes were listed in both groups. The driver genes of HNSC were identified using the “maftool” R package. The total number of nonsynonymous mutations was selected as the representative to calculate the total mutational burden of the tumors.

### Separate Patients Into Positive or Negative plac1 Expression in the TCGA-HNSC Cohort

Patients were ordered by plac1 expression, and several quantile gradients (20, 25, 33, 50, 66, 75, and 80%) were set to separate the patients. According to the Kaplan–Meier (K–M) survival analysis between the high- or low-plac1 groups, the ratio 2:1 had the best overall survival (OS) (log-rank test, *p* < 0.05). This met the truth that plac1 was highly expressed in TME subtype (D and F), which accounted for 61%. Then, we defined the upper two-third expression level as plac1-positive and the lower one-third expression level as plac1-negative.

### Functional Characterization of Differential Expression Analysis

For the RNA-seq data, edgeR and DEseq2 R packages were used. For the microarray data, R “limma” package was used to detect differential expression. Genes with *p <*0.05 and absolute fold change >1.5 were considered as differentially expressed.

The Kyoto Encyclopedia of Genes and Genomes (KEGG) database and Gene Ontology (GO) category database were used for the functional annotation of differentially expressed genes. An enrichment analysis of GO categories was performed by R clusterProfiler (v3.14.3) package, and an enrichment analysis of pathways was tested upon hypergeometric distribution by R “phyper” function. Those GO categories with a false discovery rate <0.05 were considered as significantly enriched, while pathways with *p <*0.05 were regarded as enriched. Only those GO categories or pathways containing ≥5 differentially expressed genes (DEGs) were kept for further analysis.

### Gene Set Enrichment Analysis

Gene Set Enrichment Analysis (GSEA) was performed to calculate the enrichment score of annotated pathways using R package “fgsea” (13). The genes were ranked by log_2_ (fold change). The annotated gene set included KEGG, and biological process signature and hallmark genesets were obtained from the Molecular Signatures Database (MSigDB, V7.2) (14). The immunologic signature was downloaded from Immport database (15). Gene sets with *p <*0.05 and absolute normalized enrichment score ≥ 1 was considered significantly enriched.

### Transcriptome Deconvolution of the TIME

The abundance of infiltrating immune cell populations was estimated by several deconvolution methods like MCP (16), CIBERSORT (17), and TIMER XCELL (18). All these methods were integrated in R (immunedeconv). The R package “ESTIMATE” was used for immune score, tumor purity, and stromal score for tumor samples (19).. Other immune- or tumor-associated signatures in each sample were quantified by single-sample GSEA with the R package “GSVA”. The differential enrichment level of each signature between subtypes within each tumor were detected by R “limma” package.

### Plac1-Impacted Cell–Cell Communication

Single-cell RNA-seq data for HNSC were collected from GSE103322. Malignant cell clusters with a percentage of plac1-expressing cells greater than 10% were defined as plac1-positive and others as negative. In this way, all malignant cells were classified into plac1-positive and plac1-negative cells, respectively. Samples with less than 1% of plac1-negative malignant cells were classified into plac1-positive samples and others into plac1-negative samples. Cell–cell communication was analyzed by CellChat (20).

### Plac1-Related Immune Risk Model

We calculated the Pearson correlation between plac1 expression and immunoinhibitory- or immunostimulatory-related genes in the TCGA-HNSC dataset. Genes with a correlation test *p*-value <0.05 were considered as plac1 co-expression immune genes. These genes were further used in step-wise bilateral Cox-ph regression analysis. After this, 15 genes were kept for the risk score model construction (IL10RB, LTA, BTLA, CSF1R, TIGIT, LGALS9, ICOS, CTLA4, ENTPD1, HAVCR2, IL2RA, CD244, CD80, CD86, and TNFSF13B).

### Statistical Analysis

Hierarchical clustering analysis was performed on the R “hclust” function using the complete method to identify the number of clusters in TCGA-HNSC based on the expression pattern of signatures. Univariate and multivariate Cox proportional hazard regression models were used to assess the association between the signatures and overall survival with/without clinical variables. The hazard ratio (HR) and 95% confidence interval (CI) were calculated. One-tailed or two-tailed Wilcoxon rank-sum or Student’s tests were used to compare the two groups. For comparisons of more than two groups, one-way ANOVA tests and Kruskal–Wallis tests were utilized as parametric and nonparametric methods, respectively. If not noted, there is no statistical significance in one-by-one comparison. The Kaplan–Meier method and log-rank test were conducted to compare the survival differences between the two tumor groups. Receiver operating characteristic (ROC) curves were used to evaluate the predictive performance for the response to immunotherapy, and the area under the curve (AUC) was calculated. All statistical analyses were performed using R/Bioconductor (version 3.6.1). The main R packages used are summarized in **Supplementary Table S2**.

## Results

### Specific Expression of Plac1 in Malignant Cells of HNSC

**Figure 1:**
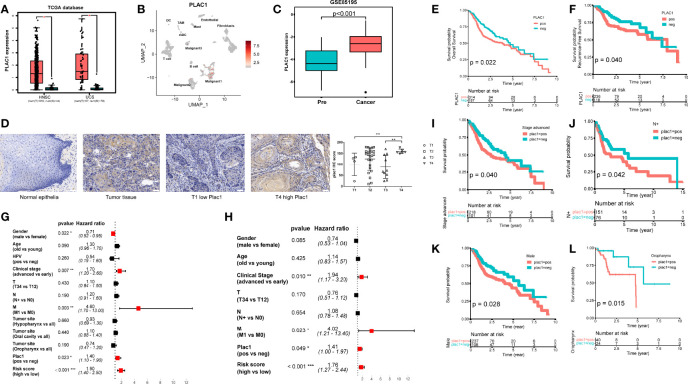
Plac1 expression patterns. **(A)** The expression status of plac1 in tumor and normal tissues in head and neck squamous cell carcinoma (HNSC) and uterine carcinosarcoma. **(B)** The plac1-positive cell distribution pattern in HNSC based on single-cell sequencing data from GSE103322. **(C)** The plac1 expression status in pre-cancer and cancer stage during HNSC tumorigenesis based on data from GSE85195. **(D)** Images of immunohistochemistry (IHC) staining for plac1 in normal epithelia tissues and tumor tissues with different T stages. Scale bars: 50 um. The Plac1 IHC score is higher in T4 stage compared to those in other T stages (right). **(E, F)** Correlation between plac1 expression and survival prognosis in The Cancer Genome Atlas-HNSC database. The survival probability of overall survival (OS) **(E)** and recurrence-free survival **(F)**, respectively, are shown. If not mentioned specifically, survival probability represents overall survival. **(G, H)** Univariate **(G)** and multivariate **(H)** Cox regression analysis of HNSC for clinical factors. **(I–L)** Correlation between plac1 expression and OS in advance-stage cancer patients **(I)**, lymph node metastasis patients **(J)**, male patients **(K)**, and oropharynx cancer patients **(L)**, respectively. *p < 0.05, **p < 0.01, ***p < 0.001.

CTAs are known for their restricted expression pattern in germline cells and cancer, which was further validated by the analysis results of mRNA expression data from TCGA database (21). As shown in **Figure 1A**, plac1 was highly expressed in tumor tissues compared to normal tissues in HNSC and uterine carcinosarcoma (also shown in **Supplementary Figures S2A, D, E**).

Given that the single-cell sequencing technique provides a single-cell resolution of gene expression patterns, we browsed the mRNA expression landscape of plac1 in the single-cell database TISCH (http://tisch.comp-genomics.org/) (22) (**Supplementary Figure S2B**). The significantly high and specific expression of plac1 in malignant cells in HNSC drew our attention. To further study which cell clusters express plac1, we used single-cell RNA sequencing data (GSE103322), conducted Uniform Manifold Approximation and Projection analysis, and labeled cells based on plac1 expression levels (**Figure 1B** and **Supplementary Figure S2C**). Considering the tumor-promoting potential of plac1 reported in colorectal cancer (10), lung cancer (7), breast cancer (11), and some other cancers, we hypothesized that this cell cluster might play an important role in oncogenesis. Thus, we examined GSE30784 and GSE85195, which are datasets composed of samples in different pathological phases during HNSC oncogenesis (**Figure 1C** and **Supplementary Figure S2D**). Significantly, plac1 expression was higher in the cancer than the precancer group (*t*-test, *p* < 0.001). The expression pattern of plac1 could also be validated in HNSC tissues and normal epithelial tissues (**Figure 1D**). We found that plac1 was negative in normal epithelia tissues while positive in tumor tissues and was more highly expressed in T4 stage tissues than in T1 and T3 stages (*t*-test, T4 *vs*. T1, *p* = 0.022, T4 *vs*. T3, *p* = 0.006).

Besides this, the distinct expression of plac1 in tumor made us wonder if this expression pattern was associated with a genetic alteration. However, the alteration frequency of plac1 in the plac1-positive group was actually low, and the alteration frequency of the major driver genes showed no significant differences between the plac1-positive and plac1-negative groups, which indicated that the regulation mechanism was beyond genetic alteration (**Supplementary Figure S3**).

These results suggested that plac1 was highly expressed in tumor tissues and may have a special tumor-promoting significance in head and neck cancer.

### The Prognostic Significance of plac1 in HNSC

The clinical significance of plac1 was supported by the survival analysis results using TCGA-HNSC data. As mentioned in methods, we adjusted the expression cutoff to the lower third, and we calculated the outcomes of HNSC patients in plac1-positive and plac1-negative groups. As shown in **Figures 1E, F**, plac1 expression was linked to poorer OS (*p* = 0.022) and relapse-free survival (*p* = 0.040). There was a tendency of the plac1-positive group toward poor disease-free survival (*p* = 0.071) and metastasis-free survival (*p* = 0.058), although the difference was not statistically significant (**Supplementary Figures S4A, B**). Moreover, the univariate and multivariate Cox regression analyses of several clinical parameters showed that, in univariate Cox regression, the HR of plac1 was 1.4, with 95% CI of 1.1–1.9 (*p* = 0.023), and in multivariate Cox regression, the HR of plac1 was 1.41, with 95% CI of 1–1.97 (*p* = 0.049) (**Figures 1G, H**). Therefore, plac1 could represent an independent risk factor for HNSC.

Cox regression also showed that several other clinical factors may affect outcomes, such as sex and clinical stage. Next, we stratified the samples by several clinical factors and estimated the correlation of the plac1 expression level with patient prognosis. In advance-stage lymph node metastasis patients, male patients, and oropharynx patients, plac1-positive patients had poorer outcomes (*p* = 0.040, 0.042, 0.028, and 0.015, respectively) (**Figures 1I–L**, **Supplementary Figures S4C–G**). Given that oropharynx cancer is related to human papillomavirus (HPV) infection, we evaluated the relationship between plac1 expression and HPV status. The results showed no correlation between these two factors. In addition, no correlation in OS was noted between HPV-positive and HPV-negative patients among plac1-positive patients (**Supplementary Figures S4H–J**). However, we found that the immune score of oropharynx cancer was higher than that of hypopharynx and oral cancer (ANOVA test, *p* = 0.031, hypopharynx *vs*. oropharynx: *p* = 0.009, oral cavity *vs*. oropharynx: *p* = 0.042) (**Supplementary Figure S4K**), which may partially explain the superior outcomes in oropharynx patients.

Epidermal growth factor receptor (EGFR) is a classical target in head and neck cancer, and we found that the anti-EGFR therapy signature was enriched in the plac1-positive group (*p* < 0.001) (**Supplementary Figures S5A,B**). The EGFR expression was also higher in the plac1-positive group (*p* = 0.016) (**Supplementary Figure S5C**). To validate this prediction, we analyzed databases and confirmed that the cetuximab (EGFR antibody) responder group had higher plac1 expression levels than nonresponders (**Supplementary Figure S5D**) (*p* = 0.048), which suggested that plac1 could be a biomarker in EGFR antibody therapy.

In summary, high plac1 expression levels correlated with poorer clinical prognosis but better anti-EGFR therapy response in head and neck cancer.

### Plac1 Signified a More Invasive Biological Behavior of HNSC

**Figure 2:**
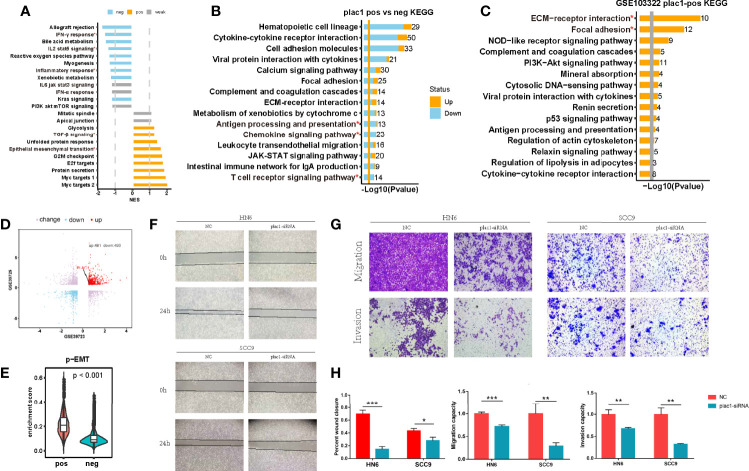
Plac1 was related to the invasive biological characteristics of head and neck squamous cell carcinoma (HNSC). **(A, B)** Gene Set Enrichment Analysis of hallmarks **(A)** and Kyoto Encyclopedia of Genes and Genomes (KEGG) analysis **(B)** in plac1-positive and plac1-negative samples from The Cancer Genome Atlas-HNSC database. **(C)** KEGG pathway gene sets in plac1-positive cells from the single-cell dataset GSE103322. **(D)** Plac1 was among the upregulated genes according to the invasion selection assay in both GSE39723 and GSE39725. **(E)** Difference in the enrichment score of the p-EMT signature in plac1-positive and plac1-negative cells from the single-cell dataset GSE103322. **(F–H)** Images of wound healing assay **(F)** and Transwell assays for migration and invasion **(G)** in negative control and plac1 knockdown group. The quantitative analysis of wound closure, migration ability, and invasion ability is shown in **(H)**. *p < 0.05, **p < 0.01, ***p < 0.001.

We were interested in the specific function of plac1 during cancer progression. We noted that TGF-β signaling and epithelial–mesenchymal transition (EMT) were upregulated in the plac1-positive group (**Figure 2A**). Based on the KEGG analysis (**Figure 2B**), cytokine–cytokine receptor interaction, chemokine signaling pathway, antigen processing and presentation, and the T cell receptor signaling pathway were negatively related to plac1. Referring to the single-cell RNA dataset (**Figure 2C**), we found that extracellular matrix–receptor interaction, focal adhesion, and other pathways were regulated in plac1-positive cells, whereas multiple metabolic pathways were downregulated, including glutathione metabolism and biosynthesis of amino acids (**Supplementary Figures S6A, B**).

Chang et al. (23) isolated a highly invasive subpopulation of HNSC cell lines using the Transwell invasion assay (GSE39725 and GSE39723). Consistent with the GO and KEGG analysis results, plac1 was highly expressed in invasive cells compared with the original cells (**Figure 2D**). Consistently, the p-EMT program, which is expressed at the invasive edge of HNSC tumor tissues (24), was upregulated in plac1-positive cells (*p* < 0.001) (**Figure 2E**). These results showing that plac1-positive cells exhibit more invasive malignant phenotypes were confirmed in wound healing assay and Transwell assays (**Figures 2F–H**). A wound healing assay showed that the knockdown of plac1 repressed the cell migratory ability. Transwell assays, including migration and invasion assays, indicated that the migratory ability as well as invasive ability were reduced after plac1 depletion.

All of the abovementioned results demonstrated that plac1-positive cells played important roles during oncogenesis and cancer progression in HNSC.

### Plac1 Was Associated With the Noninflamed Tumor Microenvironment in HNSC

**Figure 3:**
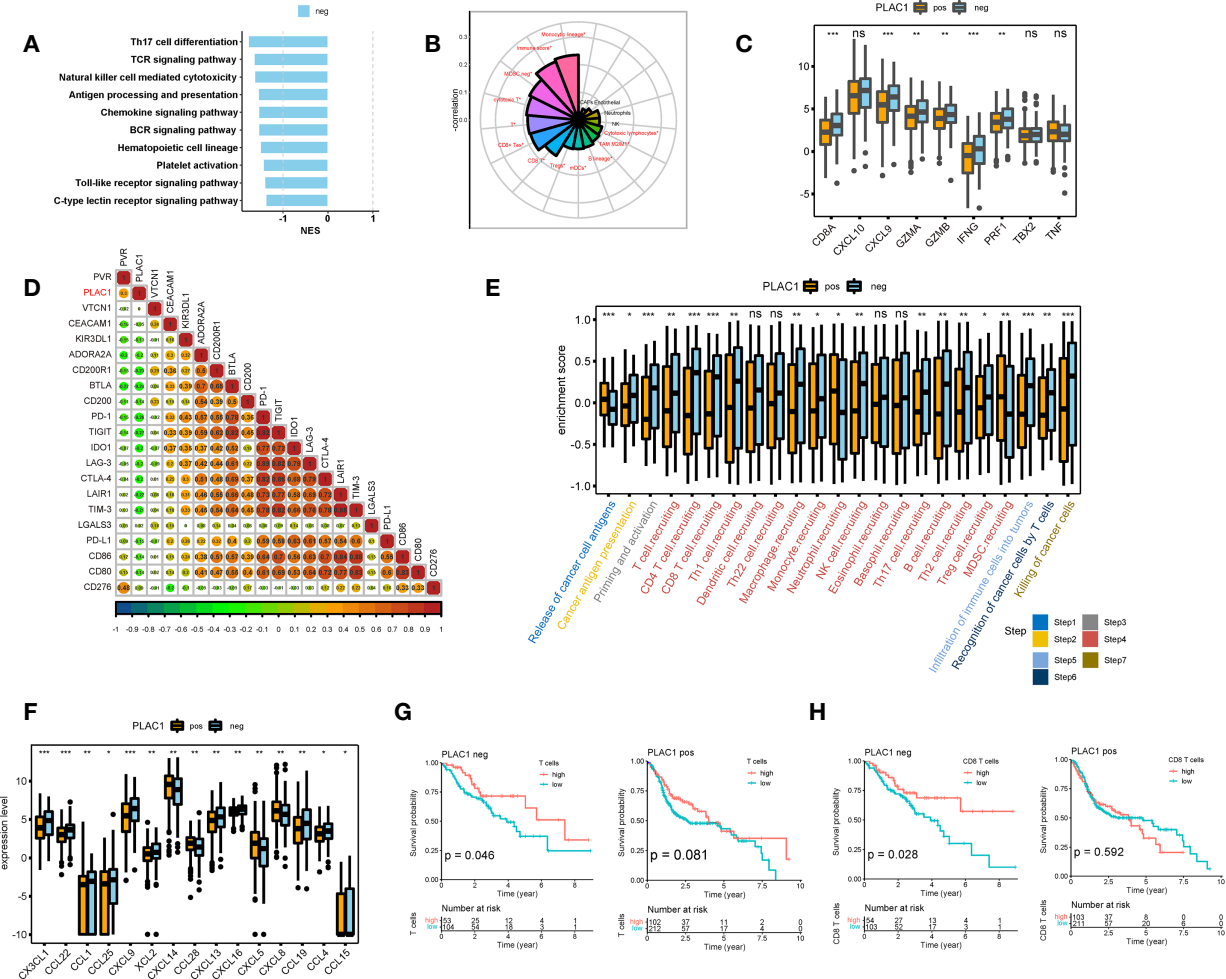
Plac1 was negatively related to the immune activities in head and neck squamous cell carcinoma. **(A)** Gene Set Enrichment Analysis of immune-related hallmarks in differentially expressed genes between plac1-positive and plac1-negative samples. **(B)** Correlations between plac1 and immune infiltration level. **(C–F)** Differences in the enrichment score or expression level of immune activity-related genes **(C)**, immune checkpoints **(D)**, immune cycles **(E)**, and chemokines **(F)** in plac1-positive and plac1-negative samples. **(G, H)** Correlations between T cell **(G)** and CD8^+^ T cell **(H)** level and patient survival in plac1-positive and plac1-negative samples. *p < 0.05, **p < 0.01, ***p < 0.001.

GSEA suggested that multiple immune pathways, including immune cell differentiation and immune receptor signaling, were downregulated in plac1-positive cells (**Figure 3A**; **Supplementary Figure S8A**). Therefore, we further examined the influence of plac1 on the immune system. For the most important immune cells, such as CD8^+^ T cells, plac1 was negatively correlated with infiltration in five different algorithms. The negative association between immune score and plac1 again confirmed this result (**Figure 3B**, **Supplementary Figures S8B–D**) (25–31). The other two main types of innate immune cells—ILC1s and ILC3s—were also negatively related to plac1 (**Supplementary Figure S7**), further supporting the abovementioned results.

However, given the complexity of the TME, the immune infiltration level is insufficient to delineate the immune function of plac1, so we examined our data with an immune-activity gene signature (**Figure 3C**), immune checkpoint gene signature (**Figure 3D**; **Supplementary Figure S8G**), immunomodulatory gene signature (**Supplementary Figure S8E**), and immune effector gene signature (**Supplementary Figure S8F**) (32). The expression level of plac1 was negatively related to immunostimulator, major histocompatibility complex, receptor, and immune-activity gene signatures. More specifically, the immune activity-related genes that showed a strong negative relevance included CD8A, CXCL9, GZMA, GZMB, IFNG, and PRF1. These genes are essential for T cell infiltration and cytotoxicity.

To further elucidate the immune functions of plac1, we performed an immune cycle analysis (33). In the plac1-positive group, the activities of the majority of the steps in the cycle were downregulated (**Figure 3E**), including T cell priming and activation (step 3), immune cell recruitment (step 4), and infiltration of immune cells into tumors (step 5). Subsequently, the decreased activities of these immune steps could reduce the infiltration levels of effector immune cells into the TME and induce subsequent steps given that the recognition of cancer cells by T cells (step 6) and the killing of cancer cells (step 8) were also downregulated. As shown in **Figure 3F**, the downregulation of a number of chemokines could damage immune infiltration in the TME, leading to the poor stimulation and functions of immune cells.

To further estimate which cell subtypes were affected by plac1-positive tumor cells, we conducted a survival analysis according to immune cell gene signatures in the plac1-positive and plac1-negative groups. As shown in **Figures 3G, H** and **Supplementary Figure S9A**, the immune score (*p* = 0.022), T cells (*p* = 0.046), and CD8^+^ T cells (*p* = 0.028) were favorable prognostic factors in the plac1-negative group. However, in the plac1-positive group, these factors lost their positive correlation with patient survival (*p* = 0.980, 0.081, and 0.592, respectively), indicating that plac1 expression negatively affects the beneficial associations among the immune score, T cells, CD8^+^ T cells, and overall survival. Next, we stratified the samples by immune infiltration and calculated the K–M curve based on plac1 expression (**Supplementary Figure S9B**). The adverse impact of plac1 was observed in the T cell-low groups (*p* = 0.008) but not in the T cell-high groups (*p* = 0.370). This finding indicated that plac1 might have a synergistic effect with low T cell infiltration.

These results showed that plac1 was associated with the noninflammed TME, so we next turned to single-cell sequencing data to figure out how plac1 reshapes the TME.

### Single-Cell Transcriptomes Depicting the Role of plac1 in Reshaping the Microenvironment in HNSC

**Figure 4:**
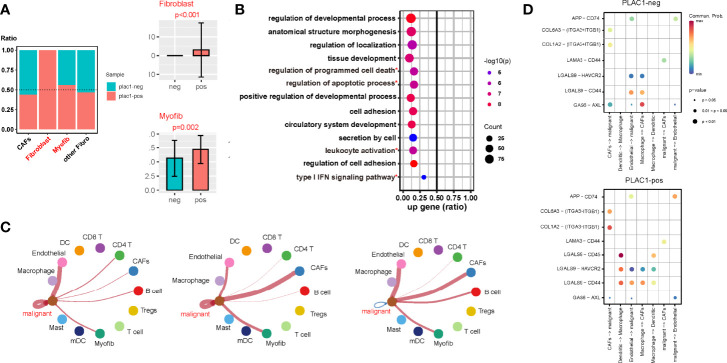
Plac1 shapes a noninflamed tumor microenvironment (TME) in head and neck squamous cell carcinoma. **(A)** Differences in the fibrotic component proportion of the TME of plac1-positive and plac1-negative samples. **(B)** Kyoto Encyclopedia of Genes and Genomes pathway analysis of fibroblasts between plac1-positive and plac1-negative samples. **(C)** Differences in the cell–cell interaction of plac1-positive and plac1-negative cells with other cells in the TME. The left panel represents cell–cell contact, the middle panel represents extracellular matrix–receptor interaction, and the right panel represents secreted signaling. **(D)** Differences in the interaction strength of specific ligand–receptor pairs between plac1-positive and plac1-negative samples. *Pathways that are concerened in further analysis.

Based on the single-cell sequencing data, we compared the TME component of plac1-positive samples with plac1-negative samples and found that fibroblasts (*p* < 0.001) and myofibroblasts (*p* = 0.002) were significantly more abundant in the plac1-positive samples (**Figure 4A**). The GO analysis of these cell types in plac1-positive samples showed a downregulation of programmed cell death and a regulation of apoptotic processes, indicating the dysregulation of cell proliferation and fiber formation of fibroblasts in plac1-positive samples. In addition, the downregulation of leukocyte activation and type I interferon (IFN) signaling pathways revealed their immunosuppressive impacts on the TME (**Figure 4B**).

The CellChat analysis of single-cell sequencing data showed that the cell–cell interaction landscape of plac1-positive samples was quite different from that of plac1-negative samples (**Supplementary Figures S10A–C**). Interactions are noted between DCs and Tregs as well as DCs and macrophages. We also compared the cell–cell interactions of plac1-positive malignant cells with plac1-negative malignant cells and found that the interactions between plac1-positive malignant cells and endothelial cells and CAFs were upregulated (**Figure 4C**). To be more specific, the ligand–receptor pair interactions are shown in **Figure 4D** and **Supplementary Figures S10D–I**. The LGALS9-CD45, LGALS9-HAVCR2, and LGALS9-CD44 interactions were strengthened in the DC–macrophage pair. APP-CD74 in the malignant–endothelial pair and COL6A3-(ITGA3 + ITGB1), COL1A2-(ITGA3 + ITGB1), and LAMA3-CD44 in the malignant–CAF pair were upregulated.

Given that we found that plac1-positive malignant cells could interact with stromal cells and enhance their interaction with each other, we hypothesized that the immunosuppressive effects of plac1 were more relevant to the stromal components than the immune components. Therefore, we calculated DEGs based on plac1 expression, immune score, and stroma score. Interestingly, fewer common genes were noted between DEGs upregulated in the plac1-positive group and immune scores than stromal scores, which supported our hypothesis (**Supplementary Figure S11**).

Combined with the abovementioned results, we concluded that plac1 shaped the noninflamed TME of HNSC by recruiting fibroblasts, forming an intensive stromal component, and preventing the infiltration of other immune cells.

### Plac1 Is a Promising Marker for Cold Tumors

**Figure 5:**
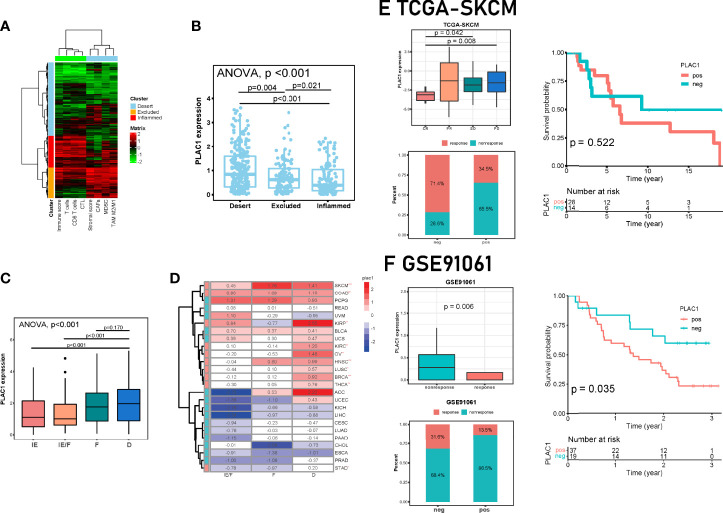
Correlations among plac1, the immune phenotype, and the clinical response to immunotherapy across cancers. **(A, B)** The Cancer Genome Atlas-head and neck squamous cell carcinoma samples were divided into three immune phenotypes based on eight gene signatures. A clustering heat map is shown in **(A)**. The differences in Plac1 expression levels in the three immune phenotypes are shown in **(B)**. Here we used one-way ANOVA test and *p <*0.001; dessert *vs*. excluded: *p* = 0.004, dessert *vs*. inflamed: *p* < 0.001; excluded *vs*. inflamed: *p* = 0.021. **(C)** Differences in the expression level of plac1 in different microenvironment (TME) phenotypes in HNSC. Here we used one-way ANOVA test and *p* < 0.001; immune-enriched/nonfibrotic (IE) *vs*. immune-depleted (D): *p* < 0.001; immune-enriched/fibrotic(IE/F) *vs*. D: *p* < 0.001; fibrotic(F) *vs*. D: *p* = 0.170. **(D)** Differences in the expression level of plac1 in different TME phenotypes in pan-cancer. Here we used *t*-test and compared between IE/F and D groups. SKCM, skin cutaneous melanoma; COAD, colon adenocarcinoma; PCPG, pheochromocytoma and paraganglioma; READ, rectum adenocarcinoma; UVM, uveal melanoma; KIRP, kidney renal papillary cell carcinoma; BLCA, bladder urothelial carcinoma; UCS, uterine carcinosarcoma; KIRC, kidney renal clear cell carcinoma; OV, ovarian serous cystadenocarcinoma; LUSC, lung squamous cell carcinoma; BRCA, breast invasive carcinoma; THCA, thyroid carcinoma; ACC, adrenocortical carcinoma; UCEC, uterine corpus endometrial carcinoma; KICH, kidney chromophobe; LIHC, liver hepatocellular carcinoma; CESC, cervical squamous cell carcinoma and endocervical adenocarcinoma; LUAD, lung adenocarcinoma; PAAD, pancreatic adenocarcinoma; CHOL, cholangiocarcinoma; ESCA, esophageal carcinoma; PRAD, prostate adenocarcinoma; STAD, stomach adenocarcinoma. **(E, F)** Correlation between plac1 and clinical response to immunotherapy. We showed differences in the expression level of plac1 in different therapy response groups, response sample counts of plac1-negative and plac1-positive groups, and correlations between plac1 expression level and patient survival. **(E)** Data from TCGA-SKCM. **(F)** Data from GSE91061. *p < 0.05, **p < 0.01, ***p < 0.001.

With the development of immunotherapy, tumors have unofficially been classified into two categories, namely, “hot” and “cold” tumors, indicating different outcomes of immunotherapies. A more comprehensive classification mode delineates the tumor immunity continuum, in which cold tumors include the excluded subtype and ignorant (desert) subtype (34). Therefore, according to the definition of cold tumors, we classified samples in the TCGA-HNSC database with several immune-related signatures (35) and investigated the plac1 gene expression levels (**Figures 5A, B**). The subtypes clustered well given that the PD-L1 expression gradient increased from the desert subtype to the excluded subtype to the inflamed subtype (**Supplementary Figure S12A**). We found that plac1 was most highly expressed in the desert subtype, and the lowest expression was noted in the inflamed subtype, again validating the analysis results mentioned above (**Figure 5B**, ANOVA test, *p* < 0.001; dessert *vs*. excluded: *p* = 0.004, dessert *vs*. inflamed: *p* < 0.001; excluded *vs*. inflamed: *p* = 0.021). Another TME subtype model delineated the TME into four subtypes: immune-enriched/nonfibrotic (IE), immune-enriched/fibrotic (IE/F), fibrotic (F), and immune-depleted (D) (36). As expected, plac1 expression was highest in the D subtype and lowest in the IE subtype (**Figure 5C**, ANOVA test, *p* < 0.001; IE *vs*. D: *p* < 0.001; IE/F *vs*. D: *p* < 0.001; F *vs*. D: *p* = 0.170). We further validated this finding in pancancer datasets and found that plac1 was highly expressed in the D subtype in SKCM, colon adenocarcinoma, kidney renal papillary cell carcinoma, kidney renal clear cell carcinoma (KIRC), ovarian serous cystadenocarcinoma, lung squamous cell carcinoma, breast invasive carcinoma, thyroid carcinoma, and stomach adenocarcinoma (**Figure 5D**, IE/F *vs*. D, *t*-test). Moreover, the plac1-positive samples showed a poorer survival in D subtypes in SKCM, KIRC and pheochromocytoma and paraganglioma (**Supplementary Figure S12B**), which consolidated the important biological function of plac1 in immune suppression. Due to the strong association between plac1 and the immune-desert phenotype, we hypothesized that plac1 is a marker of ‘cold tumor’.

### The Predictive Role of Plac1 and Novel Gene Signature in Immunotherapy Response

The immune-depleted subtype is considered to be an unfavorable factor in immunotherapy (37). The patient response to immunotherapy could be classified as response and nonresponse. Responses included complete response (CR) and partial response (PR), whereas nonresponses included stable disease (SD) and progressive disease (PD) (38). Next, we validated the strong prognostic significance of plac1 for ICB therapy in several immunotherapy cohorts (**Figures 5E, F**; **Supplementary Figures S12C, D**). In different ICB cohorts, the plac1 expression levels were significantly increased in nonresponders (PD *vs*. CR, *p* = 0.008; SD *vs*. CR, *p* = 0.042 in TCGA-SKCM, **Figure 5E**; nonresponse *vs*. response, *p* = 0.006 in GSE91061, **Figure 5F**; nonresponse *vs*. response, *p* = 0.004 in GSE126045, **Supplementary Figure S12C**; nonresponse *vs*. response, *p* = 0.035 in GSE78220, **Supplementary Figures S12D**). In addition to the therapy response status, we delineated survival curves in cohorts with survival data, and in GSE91061, the plac1-positive patients had a poorer overall survival than the plac1-negative patients (*p* = 0.035, **Figure 5F**).

Next, to optimize the prognostic efficiency of plac1, we used Pearson’s correlation analysis to generate a plac1-associated immunomodulator prognostic signature (which we defined as risk score), as shown in **Supplementary Figure S14A** and methods. The biological functions of 15 genes integrated into the signature are presented in **Table 1** (IL10RB, LTA, BTLA, CSF1R, TIGIT, LGALS9, ICOS, CTLA4, ENTPD1, HAVCR2, IL2RA, CD244, CD80, CD86, and TNFSF13B). The univariate (left) and multivariate (right) Cox regression analyses of each gene in the risk score are shown in **Supplementary Figures 13A, B**.

**Table 1:** Gene list of the 15-gene signature.

Gene symbol	Name	Function
CD86	T-lymphocyte activation antigen CD86	Receptor involved in the costimulatory signal essential for T-lymphocyte proliferation and interleukin-2 production
CD80	T-lymphocyte activation antigen CD80	Receptor induces T cell proliferation and cytokine production
CD244	NK cell type I receptor protein 2B4	Mediation of nonmajor histocompatibility complex-restricted killing
IL2RA	Interleukin 2 receptor subunit alpha	Regulation of immune tolerance by controlling TREG activity
HAVCR2	Hepatitis A virus cellular receptor 2	Regulation of macrophage activation, inhibition of Th1-mediated auto- and alloimmune responses, and promotion of immunological tolerance
ENTPD1	Ectonucleoside triphosphate diphosphohydrolase 1	Plasma membrane protein that hydrolyzes extracellular ATP and ADP into AMP
CTLA4	Cytotoxic T-lymphocyte-associated protein 4	Inhibitory receptor acting as a major negative regulator of T cell responses
ICOS	Inducible T cell costimulator	Membrane protein that enhances all basic T cell responses to a foreign antigen
LGALS9	Galectin 9	Modulation of cell–cell and cell–matrix interactions
TIGIT	T cell Immunoreceptor with Ig and ITIM domains	Assists in interactions between TFH and dendritic cells to regulate T cell-dependent B cell responses
CSF1R	Colony-stimulating factor 1 receptor	A cytokine that controls the production, differentiation, and function of macrophages
BTLA	B and T lymphocyte-associated	A receptor that relays inhibitory signals to suppress the immune response
LTA	Lymphotoxin alpha	Cytokine binding to TNFRSF1A/TNFR1, TNFRSF1B/TNFBR, and TNFRSF14/HVEM
IL10RB	Interleukin 10 receptor subunit beta	A receptor for the cytokine ligands IFNL2 and IFNL3 that mediates their antiviral activity

We first checked the risk score in the TCGA-HNSC database. The HR of the risk score was 1.90 (1.40–2.50, *p* < 0.001) in the univariate Cox regression and 1.76 (1.27–2.44, *p* < 0.001) in the multivariate Cox regression (**Figures 1F, G**), which showed that risk score could be an independent predictor of unfavorable prognosis in HNSC patients. Risk score was significantly associated with survival in HNSC, as indicated by the K–M survival curve (*p* < 0.001) and ROC curve (5-year AUC = 0.621) (**Supplementary Figure S14B**).

**Figure 6:**
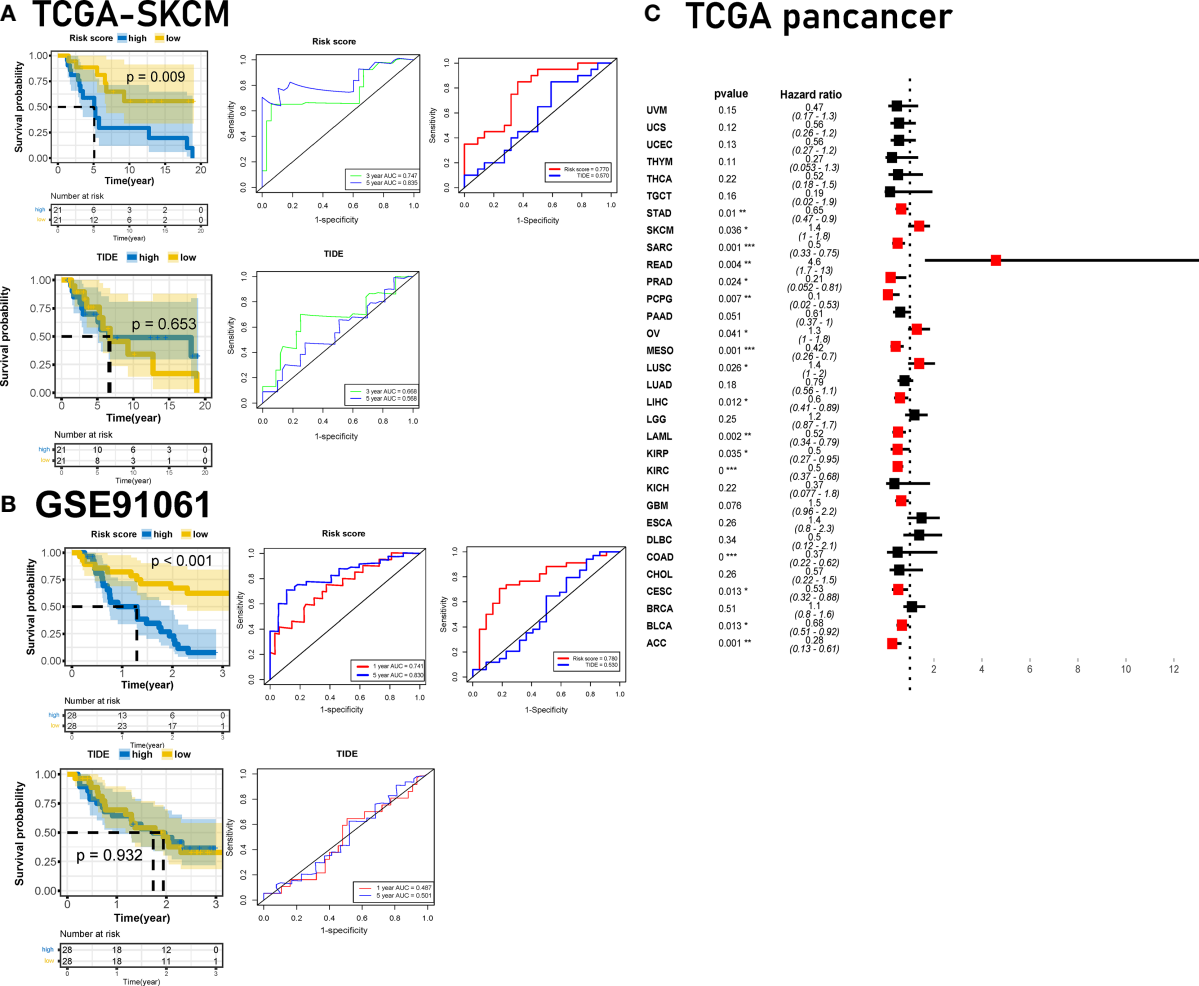
Developing the plac1-related gene risk score and validating it in immune checkpoint blocker cohorts. **(A, B)** Validation of the prognostic value of the risk score in The Cancer Genome Atlas-skin cutaneous melanoma **(A)** and GSE91061 **(B)** datasets compared with TIDE algorithms. **(C)** Univariate Cox regression analysis of pancancer for plac1. *p < 0.05, **p < 0.01, ***p < 0.001.

In addition to the general prognosis prediction, the risk score can predict the clinical response to immunotherapy and patient survival in ICB cohorts. We verified the plac1 risk score in other immunotherapy cohorts, and we compared the risk score with the TIDE traditional immunotherapy prediction model (**Figures 6A, B** and **Supplementary Figure S14C**). The results of the K–M curve analysis confirmed that patients with high plac1 risk scores had a poorer overall survival with ICB therapy (*p* = 0.009, 5-year AUC = 0.835 in the TC SGA-SKCM cohort, **Figure 6A**; *p* < 0.001, 5-year AUC = 0.830 in GSE91061, **Figure 6B**; *p* = 0.012, 5-year AUC = 0.862 in GSE78220, **Supplementary Figure S14C**), and its prediction efficiency was superior to that of TIDE (*p* = 0.653, 5-year AUC = 0.568 in the TCGA-SKCM cohort, **Figure 6A**; *p* = 0.932, 5-year AUC = 0.501 in GSE91061, **Figure 6B**; *p* = 0.260, 5-year AUC = 0.435 in GSE78220, **Supplementary Figure S14C**). Our findings demonstrated that the risk score was a valuable prognostic factor in immunotherapy cohorts.

Finally, we investigated the clinical significance of plac1 and the risk score across cancers. Plac1 was a risk factor in multiple cancers (**Figure 6C**), and the risk score worked well in different TCGA cancer cohorts (**Supplementary Figure S15**).

In general, plac1 and the risk score effectively predicted ICB response and overall survival in pancancer cohorts.

## Discussion

Cancer/testis antigens are composed of approximately 250 genes, which have a restricted expression pattern limited to germline cells and tumor cells. Due to this special expression pattern, CTAs have long been studied for cancer therapy development. Here we focused on plac1, a gene belonging to the CTA family that is highly expressed in HNSC tumor tissues. We conducted a comprehensive analysis of its expression characteristics, prognostic efficiency, biological functions, and role in shaping a noninflamed TME in HNSC. Finally, we screened for plac1-related immunomodulators and constructed a 15-gene prognostic signature, the efficiency and accuracy of which were validated in immunotherapy cohorts and a pancancer database.

Since in recent years immunotherapy has become a promising treatment for advanced cancer patients, we focused our study on the immunosuppressive impact of plac1 and its prognostic prediction value for ICB therapy.

Multiple studies have tried to conclude a perfect pipeline for predicting ICB outcomes. Traditional prognostic prediction methods are mainly based on genetic alterations, such as microsatellite instability and TMB (39, 40). However, comprehensive meta-analysis studies have shown that TMB fails to predict the outcomes in ICB therapy cohorts (41, 42). A major shortcoming of these predictors, such as TMB, is that they do not consider the complex tumor environment. Besides prediction methods on the genetic level, other signatures which are based on transcriptome data mainly focused on the immune-inflamed phenotype (43), which is composed of multiple immune cells, and the main immunosuppressive mechanism is exhaustion through immune checkpoints (35). Actually, except for immune exhaustion, immune exclusion is also an important mechanism for immune evasion of tumor cells (44). Recently, a pancancer TME subtype classification (36) drew our attention. We thought that this classification could better delineate the TME landscape than others, and the plac1-related TME belongs to the D type. Since we have validated the immunosuppressive function of plac1 by comprehensive analysis, we decided to construct a prediction signature based on this hub gene to better represent the characteristics of TME.

We developed an immune-related 15-gene signature by screening immunomodulators and selecting the top genes associated with plac1. Given that plac1 is a relatively uncharacterized gene, this process could provide a novel idea for creating prognostic models. We further validated our risk score in several immunotherapy cohorts and compared it with the TIDE classical prediction model (45). The results showed that our risk score performed better than the TIDE model. Moreover, pancancer validation showed that our method for constructing a prediction signature not only worked well in HNSC but could also be extrapolated to other cancer types.

We should also note that, with the development of single-cell sequencing technique, special subtypes of immune and stromal cells have been identified in TME from ICB samples, which performed well in predicting responses toward ICB therapy (46, 47). This could give us a new insight of prognostic prediction.

Some shortcomings of our study should be noted. Firstly, we lack experimental results to demonstrate the immunosuppressive effects of plac1 on TME. Actually, this is included as a part of our study, and we have achieved several interesting results thus far. To tell a more complete story about plac1 and antitumor immunity, we did not post our results here. Secondly, when searching for cohorts for validation, there are no available data on immunotherapy in HNSC, so we had to employ other cancer types for validation. We believe that the ongoing clinical trials on HNSC will soon provide surprising data.

## Data Availability Statement

The original contributions presented in the study are included in the article/**Supplementary Material**. Further inquiries can be directed to the corresponding author.

## Author Contributions

XM and ZL conceived the work and designed the study, and LZ helped to process data. XM wrote the original manuscript, and ZL contributed to the manuscript revision. YH supervised the study. All authors contributed to the article and approved the submitted version.

## Funding

The research activities led by YH are made possible by the funder who had no role in writing this manuscript: National Natural Science Foundation of China (no. 81900969 and no. 82173451) and Project of Biobank (no. YBKB202105) from Shanghai Ninth People’s Hospital, Shanghai Jiao Tong University School of Medicine.

## Conflict of Interest

Author LZ is employed by Suzhou Lingdian Biotechnology Co., Ltd.

The remaining authors declare that the research was conducted in the absence of any commercial or financial relationships that could be construed as a potential conflict of interest.

## Publisher’s Note

All claims expressed in this article are solely those of the authors and do not necessarily represent those of their affiliated organizations, or those of the publisher, the editors and the reviewers. Any product that may be evaluated in this article, or claim that may be made by its manufacturer, is not guaranteed or endorsed by the publisher.
