# Supplementary material for: Plac1 Remodels the Tumor Immune Evasion Microenvironment and Predicts Therapeutic Response in Head and Neck Squamous Cell Carcinoma
Source: Front Oncol. 2022 Jun 24;12:919436. doi: 10.3389/fonc.2022.919436 (PMC9263085; doi:10.3389/fonc.2022.919436)
Supplement: Supplementary file 1 [file DataSheet_1.pdf]

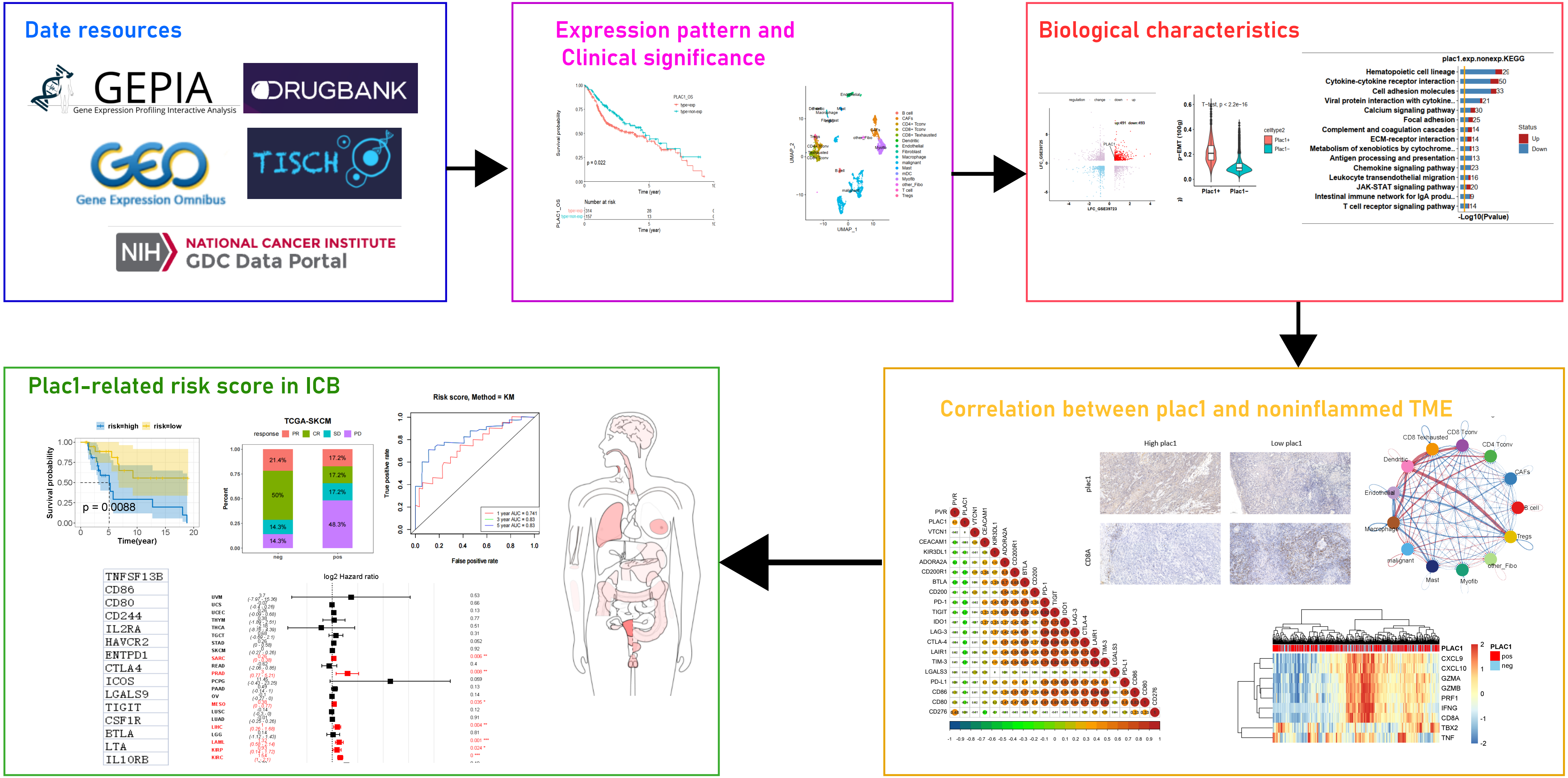

Date resources
GEPIA
Gene Expression Profiling Interactive Analysis
DRUGBANK
Gene Expression Omnibus
TISCH
NIH
NATIONAL CANCER INSTITUTE
GDC Data Portal
Expression pattern and
Clinical significance
Biological characteristics
plac1.exp.nonexp.KEGG
Hematopoietic cell lineage
Cytokine-cytokine receptor interaction
Cell adhesion molecules
Viral protein interaction with cytokine..
Calcium signaling pathway
Focal adhesion
Complement and coagulation cascades
ECM-receptor interaction
Metabolism of xenobiotics by cytochrome..
Antigen processing and presentation
Chemokine signaling pathway
Leukocyte transendothelial migration
JAK-STAT signaling pathway
Intestinal immune network for IgA produ..
T cell receptor signaling pathway
-Log10(Pvalue)
Status
Up
Down
Plac1+
Plac1−
Correlation between plac1 and noninflammed TME
High plac1
Low plac1
plac1
CD8A
Plac1-related risk score in ICB
risk=high
risk=low
Survival probability
p = 0.0088
Time(year)
TCGA-SKCM
response
PR
CR
SD
PD
Percent
neg
pos
Risk score, Method = KM
True positive rate
False positive rate
1 year AUC = 0.741
3 year AUC = 0.83
5 year AUC = 0.83
log2 Hazard ratio
TNFSF13B
CD86
CD80
CD244
IL2RA
HAVCR2
ENTPD1
CTLA4
ICOS
LGALS9
TIGIT
CSF1R
BTLA
LTA
IL10RB

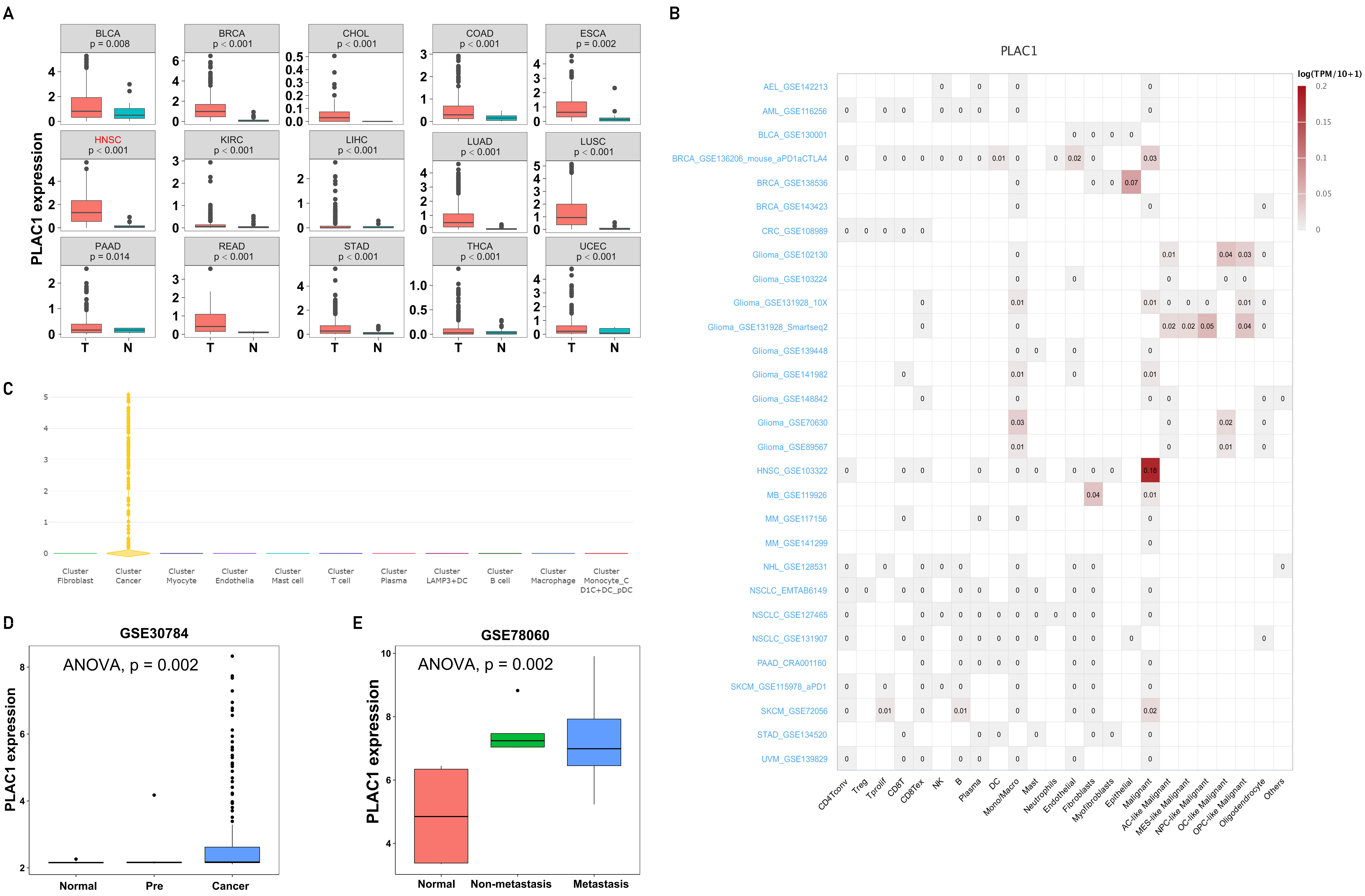
A
BLCA p = 0.008
BRCA p < 0.001
CHOL p < 0.001
COAD p < 0.001
ESCA p = 0.002
HNSC p < 0.001
KIRC p < 0.001
LIHC p < 0.001
LUAD p < 0.001
LUSC p < 0.001
PAAD p = 0.014
READ p < 0.001
STAD p < 0.001
THCA p < 0.001
UCEC p < 0.001
PLAC1 expression
T N
B
PLAC1
log(TPM/10+1)
AEL_GSE142213
AML_GSE116256
BLCA_GSE130001
BRCA_GSE136206_mouse_aPD1aCTLA4
BRCA_GSE138536
BRCA_GSE143423
CRC_GSE108989
Glioma_GSE102130
Glioma_GSE103224
Glioma_GSE131928_10X
Glioma_GSE131928_Smartseq2
Glioma_GSE139448
Glioma_GSE141982
Glioma_GSE148842
Glioma_GSE70630
Glioma_GSE89567
HNSC_GSE103322
MB_GSE119926
MM_GSE117156
MM_GSE141299
NHL_GSE128531
NSCLC_EMTAB6149
NSCLC_GSE127465
NSCLC_GSE131907
PAAD_CRA001160
SKCM_GSE115978_aPD1
SKCM_GSE72056
STAD_GSE134520
UVM_GSE139829
CD4Tconv Treg Tprolif CD8T CD8Tex NK B Plasma DC Mono/Macro Mast Neutrophils Endothelial Fibroblasts Myofibroblasts Epithelial Malignant AC-like Malignant MES-like Malignant NPC-like Malignant OC-like Malignant OPC-like Malignant Oligodendrocyte Others
C
Cluster Fibroblast
Cluster Cancer
Cluster Myocyte
Cluster Endothelia
Cluster Mast cell
Cluster T cell
Cluster Plasma
Cluster LAMP3+DC
Cluster B cell
Cluster Macrophage
Cluster Monocyte_C D1C+DC_pDC
D
GSE30784
ANOVA, p = 0.002
PLAC1 expression
Normal Pre Cancer
E
GSE78060
ANOVA, p = 0.002
PLAC1 expression
Normal Non-metastasis Metastasis

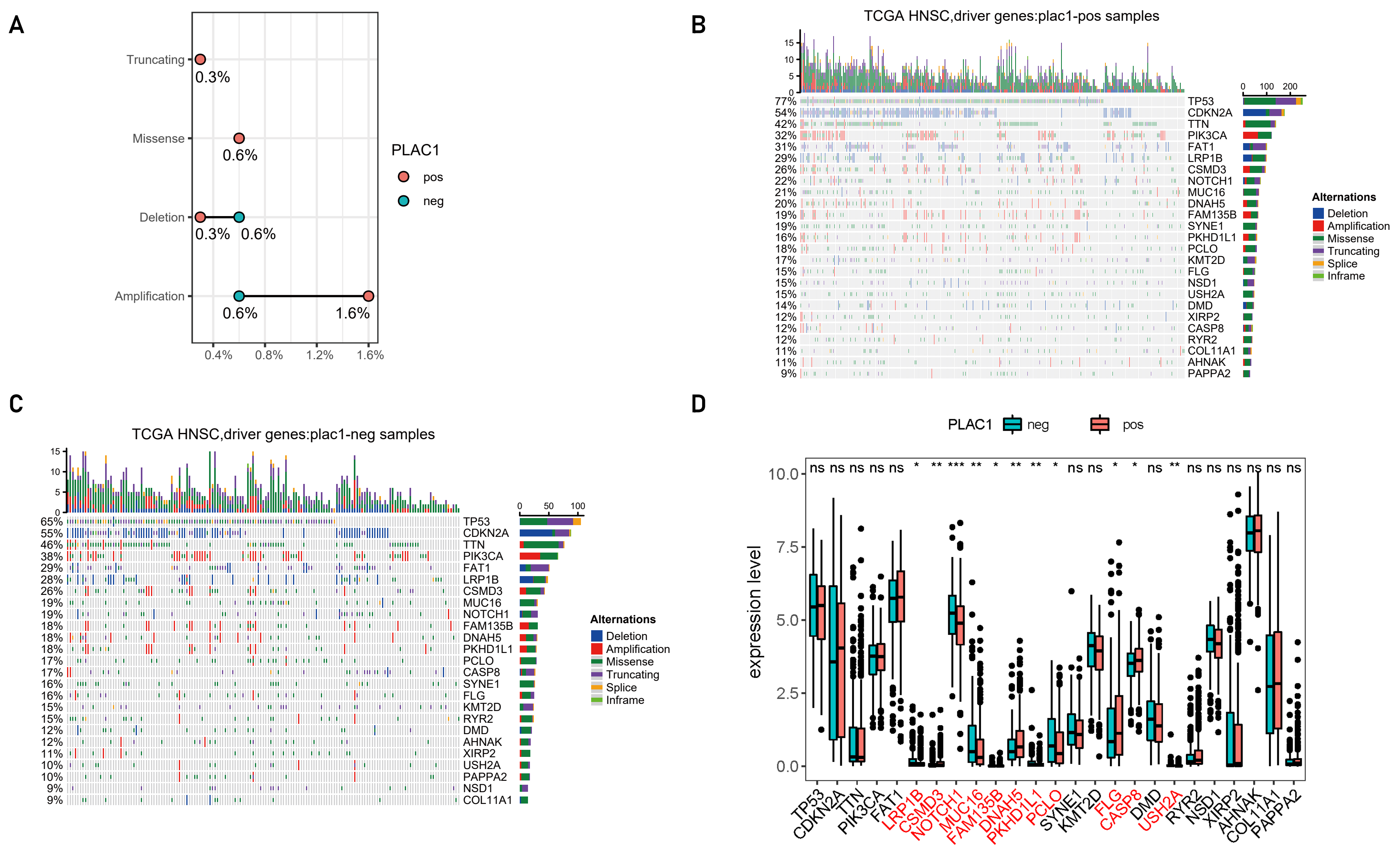
A
Truncating
Missense
Deletion
Amplification
0.3%
0.6%
0.3%
0.6%
0.6%
1.6%
0.4%
0.8%
1.2%
1.6%
PLAC1
pos
neg
B
TCGA HNSC,driver genes:plac1-pos samples
Alternations
Deletion
Amplification
Missense
Truncating
Splice
Inframe
TP53
CDKN2A
TTN
PIK3CA
FAT1
LRP1B
CSMD3
NOTCH1
MUC16
DNAH5
FAM135B
SYNE1
PKHD1L1
PCLO
KMT2D
FLG
NSD1
USH2A
DMD
XIRP2
CASP8
RYR2
COL11A1
AHNAK
PAPPA2
C
TCGA HNSC,driver genes:plac1-neg samples
D
PLAC1
neg
pos
expression level

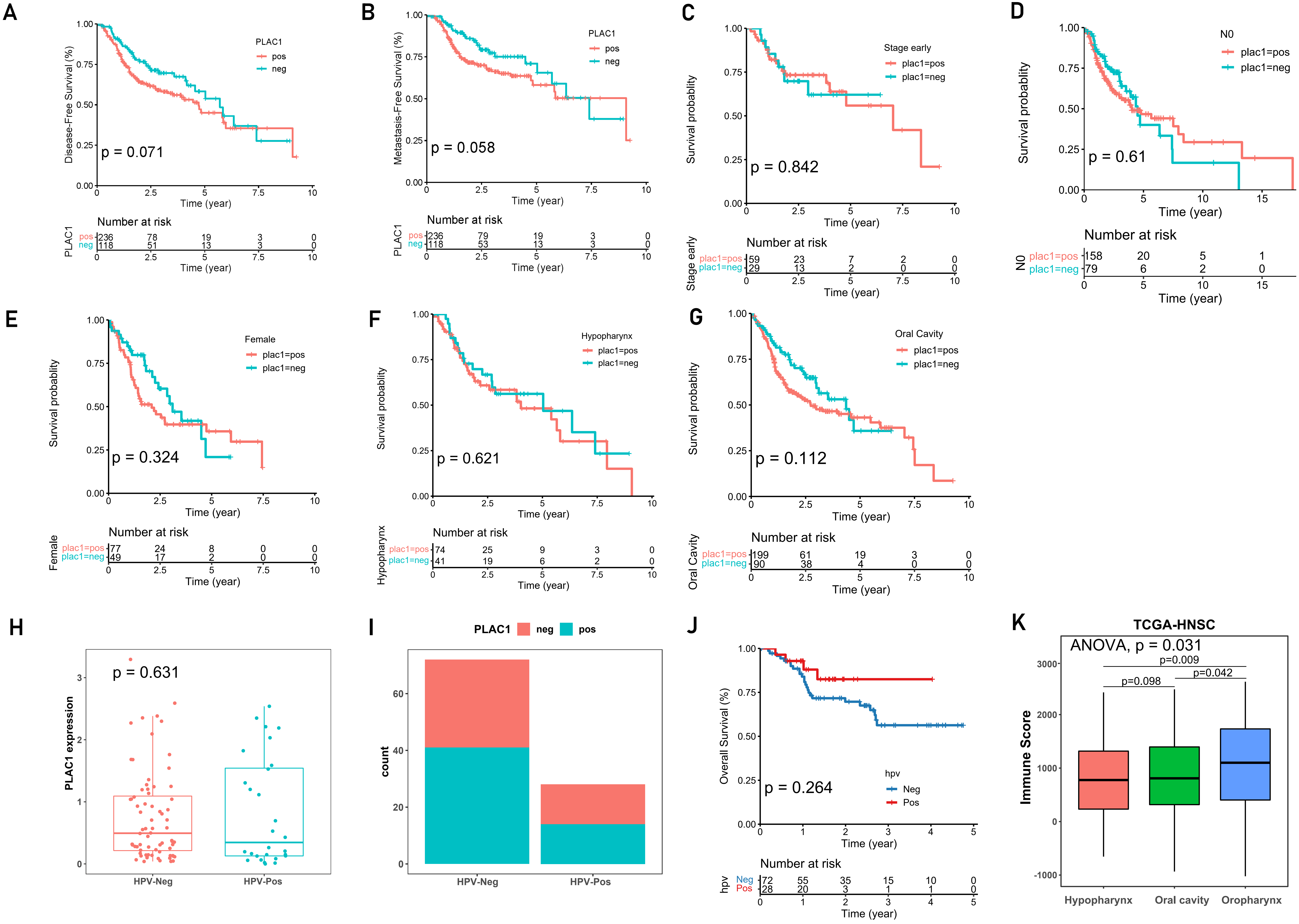
A
PLAC1
pos
neg
Disease-Free Survival (%)
p = 0.071
Time (year)
Number at risk
236 78 19 3 0
118 51 13 3 0
B
Metastasis-Free Survival (%)
p = 0.058
236 79 19 3 0
118 53 13 3 0
C
Stage early
plac1=pos
plac1=neg
Survival probability
p = 0.842
59 23 7 2 0
29 13 2 0 0
D
N0
p = 0.61
158 20 5 1
79 6 2 0
E
Female
p = 0.324
77 24 8 0 0
49 17 2 0 0
F
Hypopharynx
p = 0.621
74 25 9 3 0
41 19 6 2 0
G
Oral Cavity
p = 0.112
199 61 19 3 0
90 38 4 0 0
H
PLAC1 expression
p = 0.631
HPV-Neg
HPV-Pos
I
PLAC1 neg pos
count
HPV-Neg
HPV-Pos
J
Overall Survival (%)
p = 0.264
hpv
Neg
Pos
72 55 35 15 10 0
28 20 3 1 1 0
K
TCGA-HNSC
ANOVA, p = 0.031
p=0.009
p=0.098
p=0.042
Immune Score
Hypopharynx
Oral cavity
Oropharynx

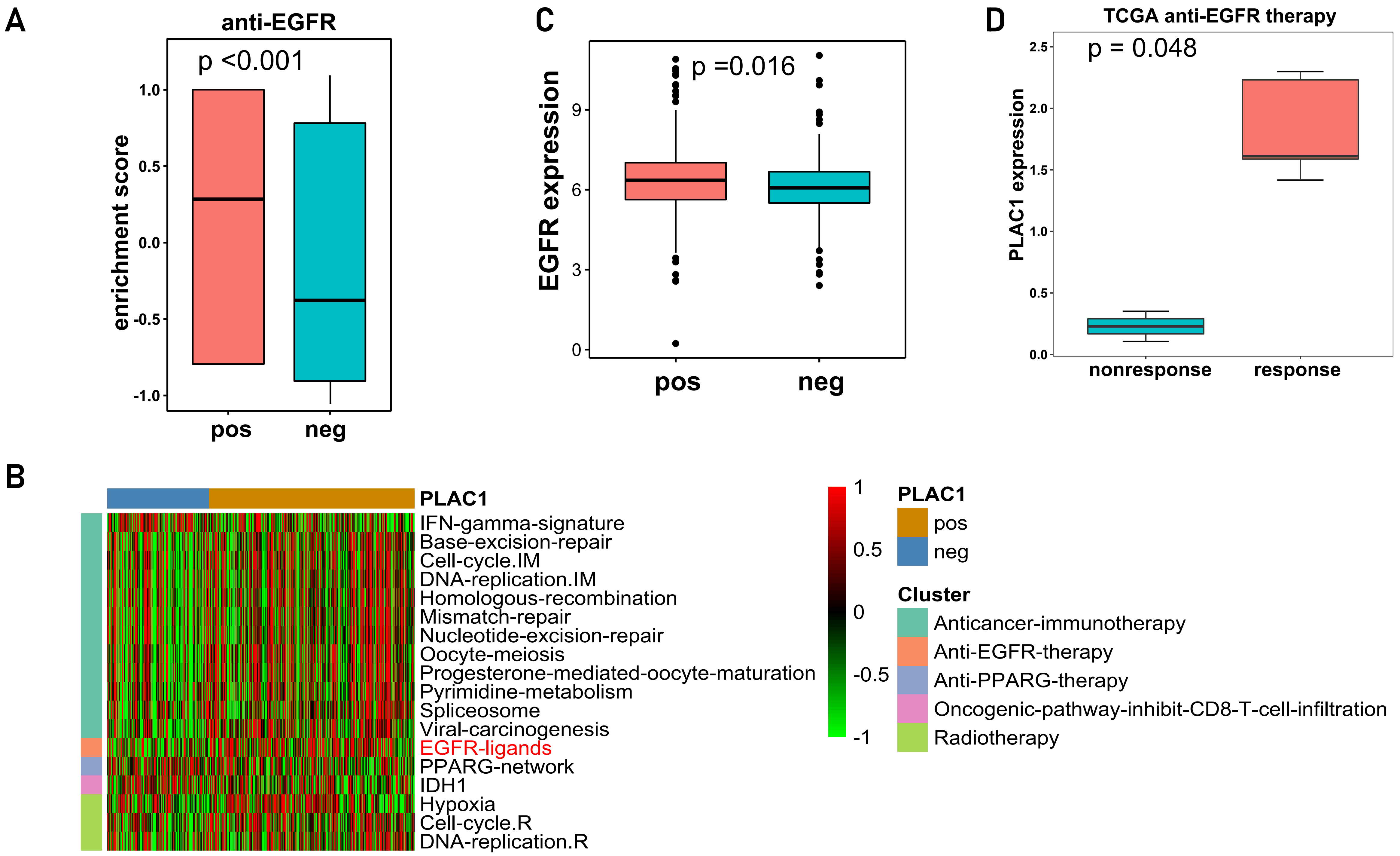
A
anti-EGFR
p <0.001
enrichment score
1.0
0.5
0.0
-0.5
-1.0
pos
neg
C
EGFR expression
p =0.016
9
6
3
0
pos
neg
D
TCGA anti-EGFR therapy
p = 0.048
PLAC1 expression
2.5
2.0
1.5
1.0
0.5
0.0
nonresponse
response
B
PLAC1
IFN-gamma-signature
Base-excision-repair
Cell-cycle.IM
DNA-replication.IM
Homologous-recombination
Mismatch-repair
Nucleotide-excision-repair
Oocyte-meiosis
Progesterone-mediated-oocyte-maturation
Pyrimidine-metabolism
Spliceosome
Viral-carcinogenesis
EGFR-ligands
PPARG-network
IDH1
Hypoxia
Cell-cycle.R
DNA-replication.R
1
0.5
0
-0.5
-1
PLAC1
pos
neg
Cluster
Anticancer-immunotherapy
Anti-EGFR-therapy
Anti-PPARG-therapy
Oncogenic-pathway-inhibit-CD8-T-cell-infiltration
Radiotherapy

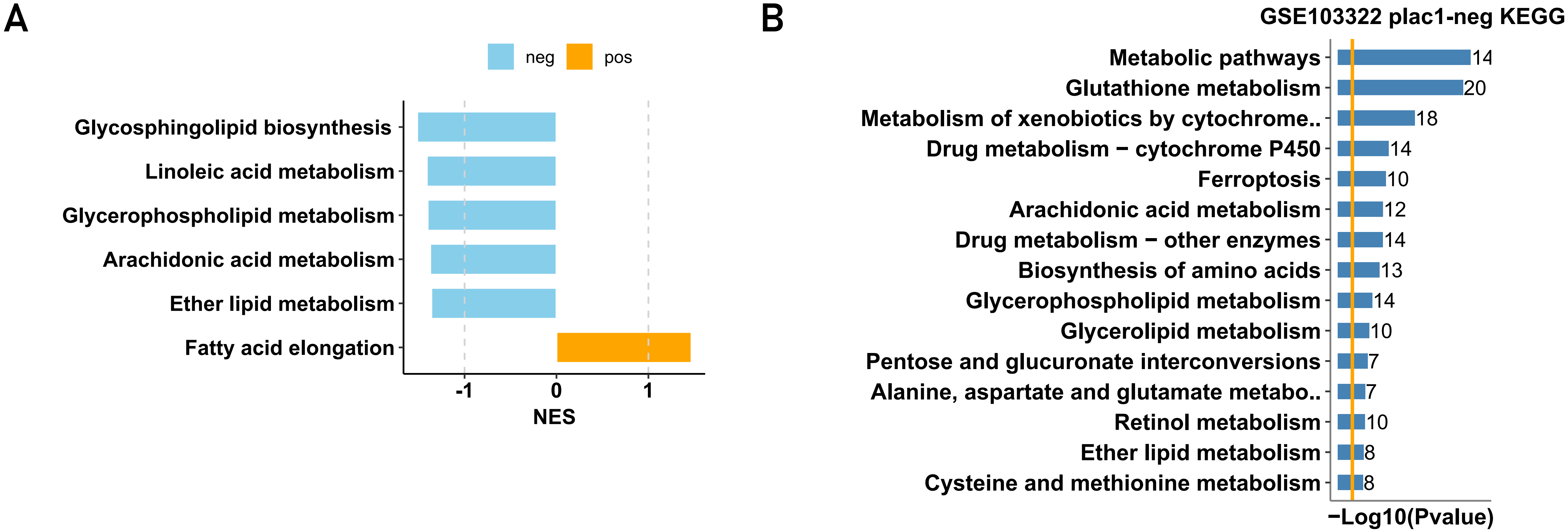
A
neg
pos
Glycosphingolipid biosynthesis
Linoleic acid metabolism
Glycerophospholipid metabolism
Arachidonic acid metabolism
Ether lipid metabolism
Fatty acid elongation
-1
0
1
NES
B
GSE103322 plac1-neg KEGG
Metabolic pathways 14
Glutathione metabolism 20
Metabolism of xenobiotics by cytochrome.. 18
Drug metabolism − cytochrome P450 14
Ferroptosis 10
Arachidonic acid metabolism 12
Drug metabolism − other enzymes 14
Biosynthesis of amino acids 13
Glycerophospholipid metabolism 14
Glycerolipid metabolism 10
Pentose and glucuronate interconversions 7
Alanine, aspartate and glutamate metabo.. 7
Retinol metabolism 10
Ether lipid metabolism 8
Cysteine and methionine metabolism 8
−Log10(Pvalue)

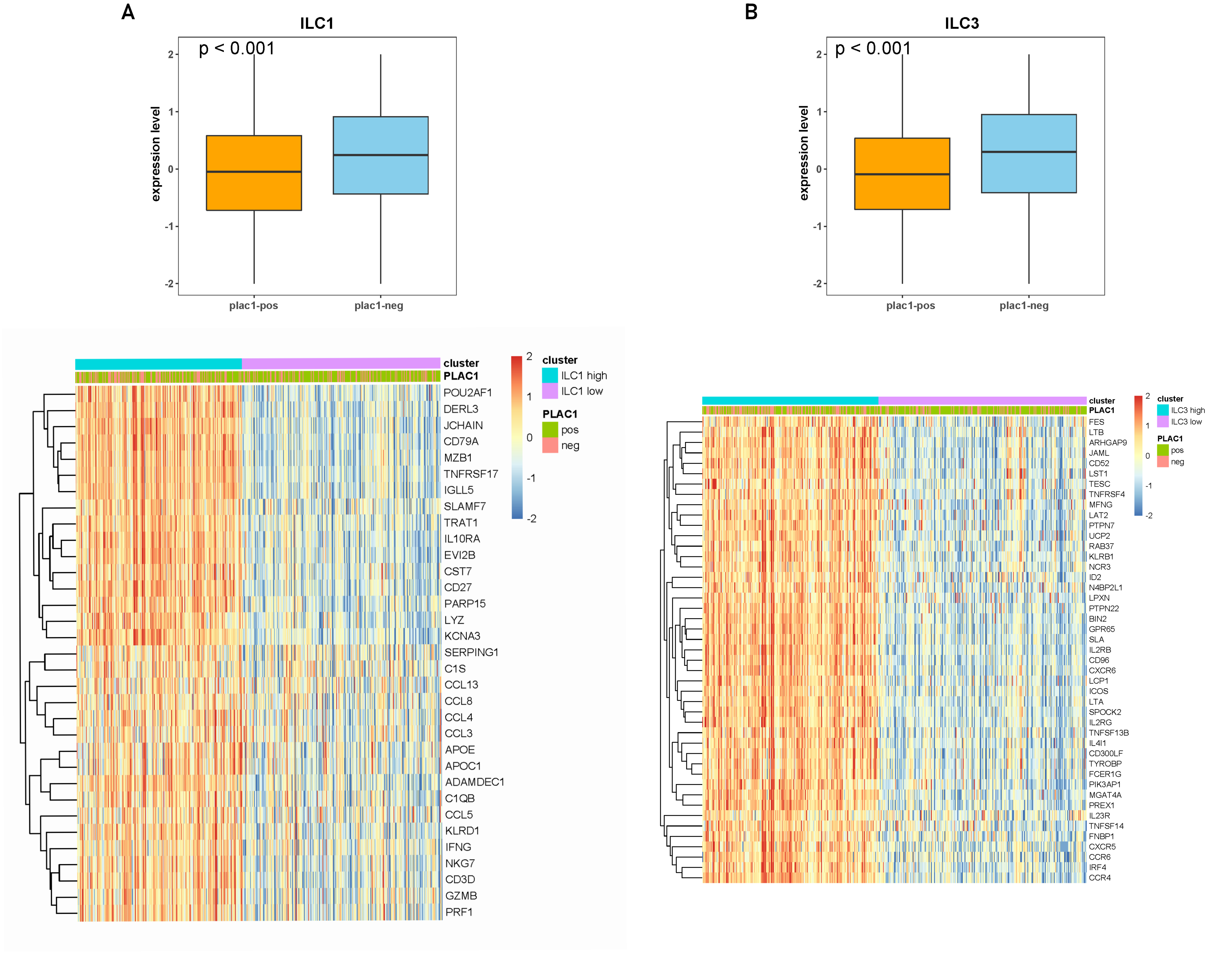
A
ILC1
p < 0.001
expression level
2
1
0
-1
-2
plac1-pos
plac1-neg
B
ILC3
p < 0.001
expression level
plac1-pos
plac1-neg
cluster
PLAC1
POU2AF1
DERL3
JCHAIN
CD79A
MZB1
TNFRSF17
IGLL5
SLAMF7
TRAT1
IL10RA
EVI2B
CST7
CD27
PARP15
LYZ
KCNA3
SERPING1
C1S
CCL13
CCL8
CCL4
CCL3
APOE
APOC1
ADAMDEC1
C1QB
CCL5
KLRD1
IFNG
NKG7
CD3D
GZMB
PRF1
cluster
ILC1 high
ILC1 low
PLAC1
pos
neg
cluster
PLAC1
FES
LTB
ARHGAP9
JAML
CD52
LST1
TESC
TNFRSF4
MFNG
LAT2
PTPN7
UCP2
RAB37
KLRB1
NCR3
ID2
N4BP2L1
LPXN
PTPN22
BIN2
GPR65
SLA
IL2RB
CD96
CXCR6
LCP1
ICOS
LTA
SPOCK2
IL2RG
TNFSF13B
IL4I1
CD300LF
TYROBP
FCER1G
PIK3AP1
MGAT4A
PREX1
IL23R
TNFSF14
FNBP1
CXCR5
CCR6
IRF4
CCR4
cluster
ILC3 high
ILC3 low
PLAC1
pos
neg

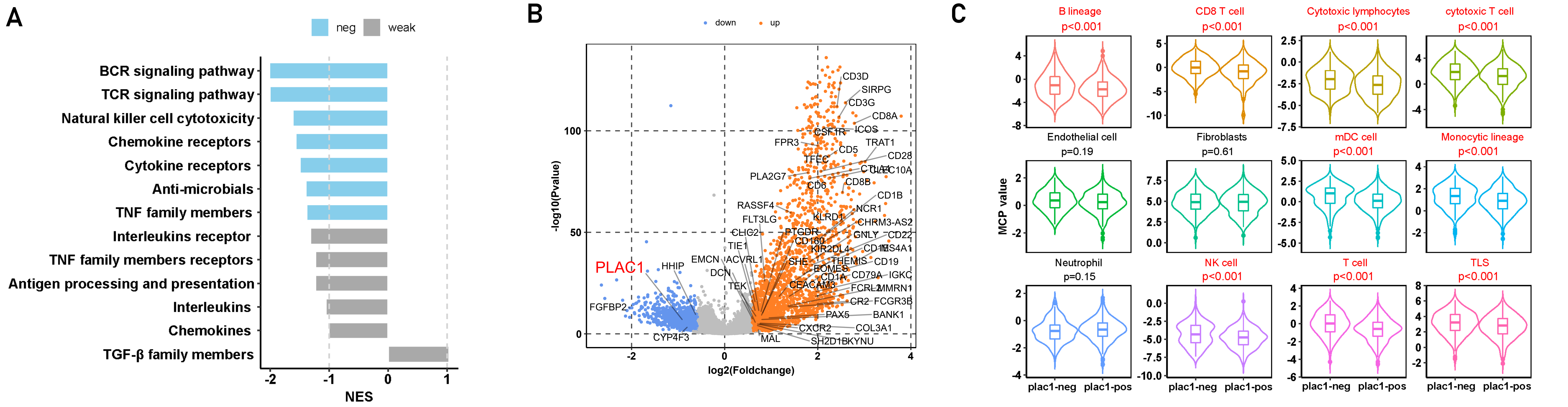

D

	MCP	Timer	Estimate	Cibersort	Cibersort 2
T	-0.6194	NA	NA	NA	NA
CD4 T	NA	-0.0011	NA	NA	0
CD8 T	-0.8067	-0.0016	NA	NA	-0.0176
Cytotoxic lymphocyte	-0.6471	NA	NA	NA	NA
DC	NA	NA	NA	-0.0053	-0.0396
B	-0.7083	-0.0016	NA	-0.0286	0.0324

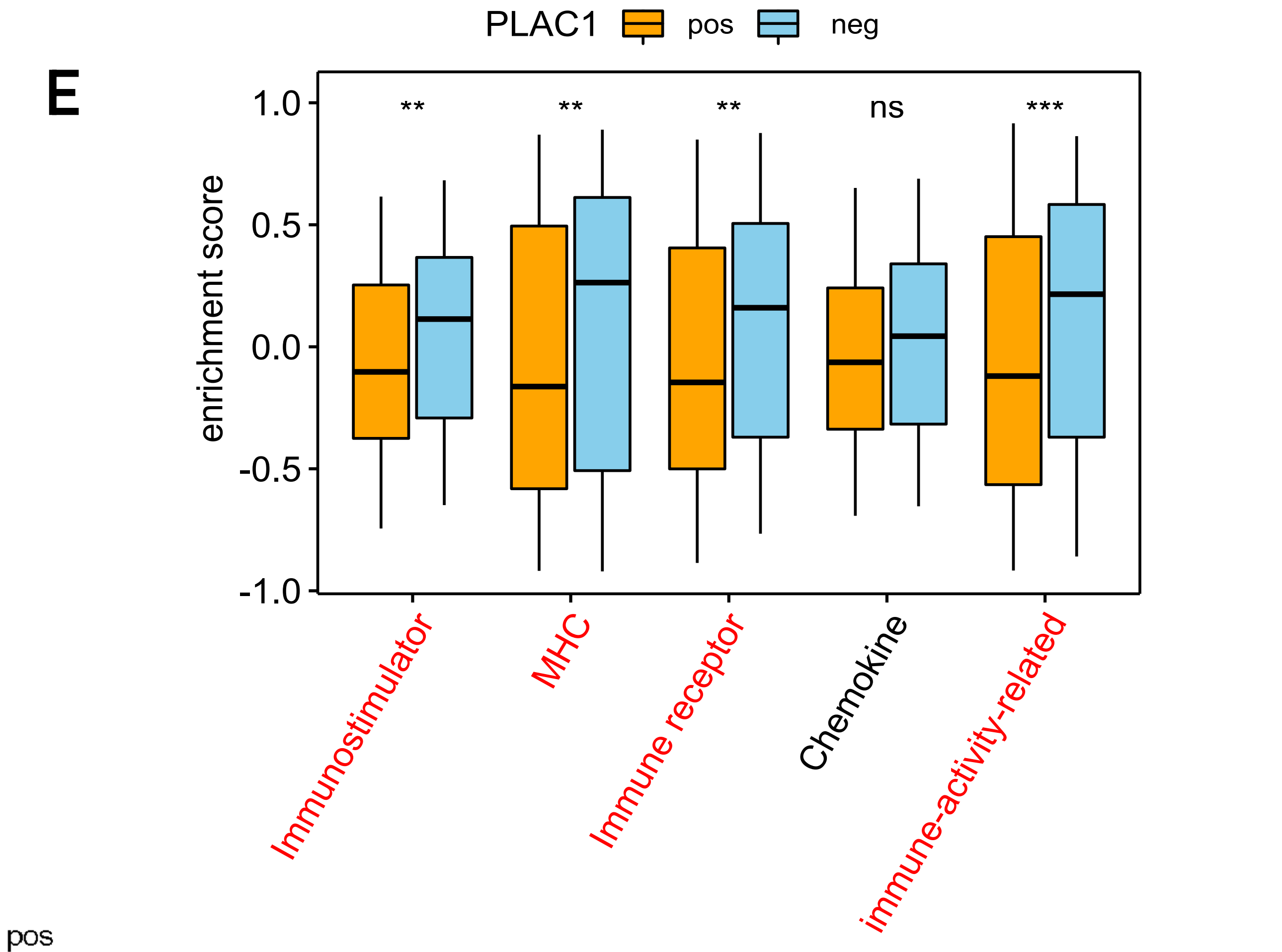

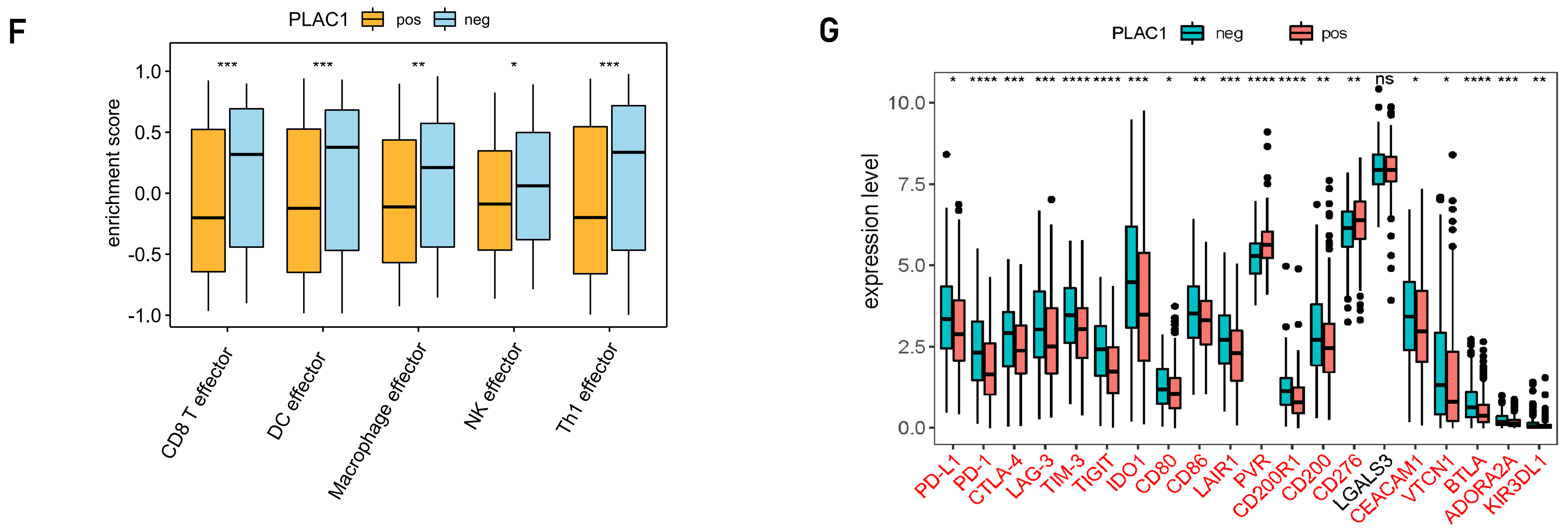

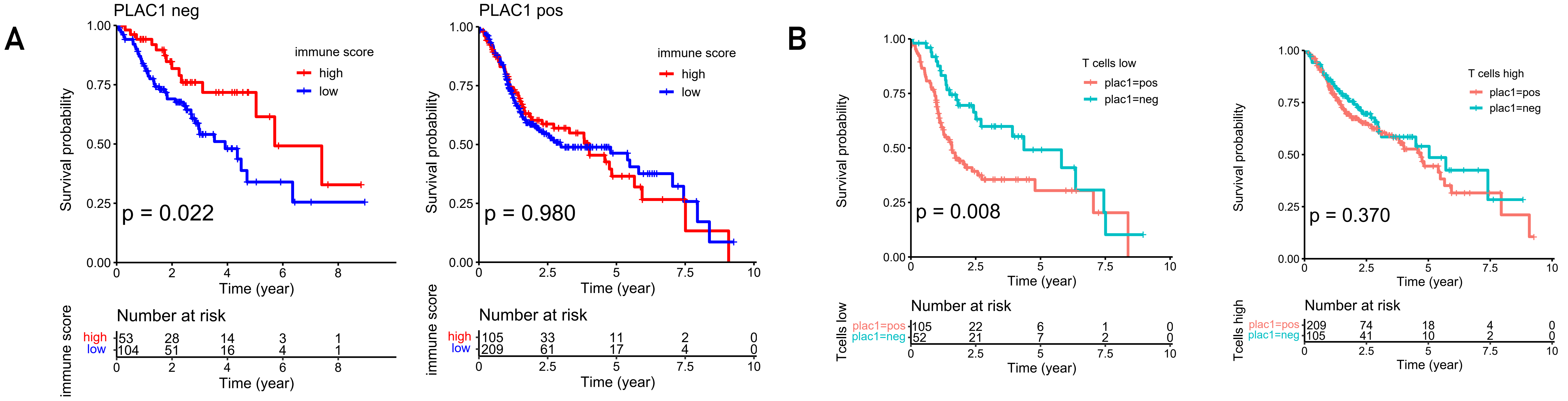

A
PLAC1 neg
immune score
high
low
Survival probability
p = 0.022
Time (year)
Number at risk
immune score
high 53 28 14 3 1
low 104 51 16 4 1
PLAC1 pos
p = 0.980
high 105 33 11 2 0
low 209 61 17 4 0
B
T cells low
plac1=pos
plac1=neg
p = 0.008
Tcells low
plac1=pos 105 22 6 1 0
plac1=neg 52 21 7 2 0
T cells high
p = 0.370
Tcells high
plac1=pos 209 74 18 4 0
plac1=neg 105 41 10 2 0

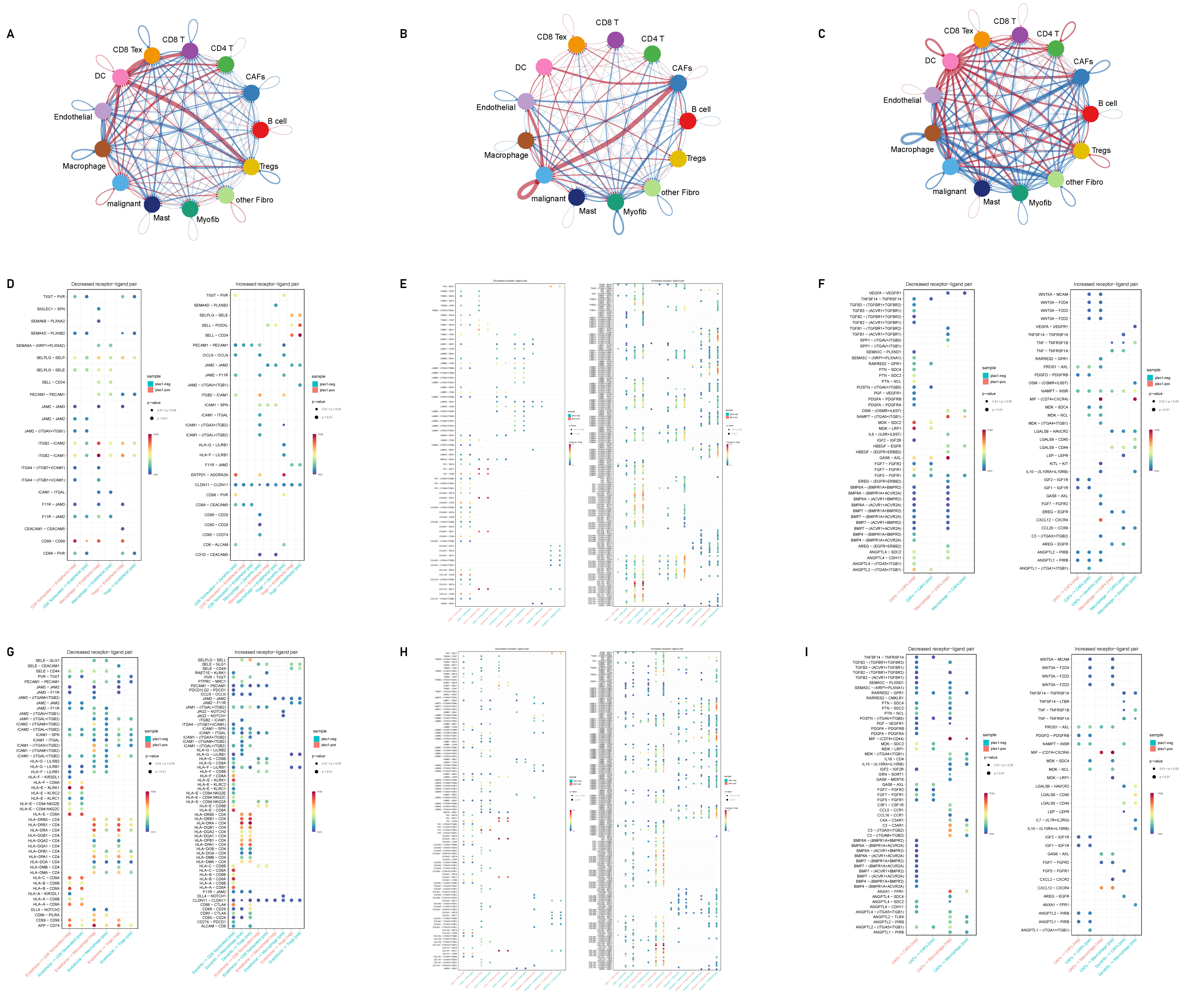
A
CD8 Tex
CD8 T
CD4 T
DC
CAFs
Endothelial
B cell
Macrophage
Tregs
malignant
Mast
Myofib
other Fibro
B
C
D
Decreased receptor-ligand pair
Increased receptor-ligand pair
TIGIT - PVR
SIGLEC1 - SPN
SEMA6B - PLXNA2
SEMA4D - PLXNB2
SEMA4A - (NRP1+PLXNA2)
SELPLG - SELP
SELPLG - SELE
SELL - CD34
PECAM1 - PECAM1
JAM2 - JAM3
JAM2 - JAM2
JAM2 - (ITGAV+ITGB1)
ITGB2 - ICAM2
ITGB2 - ICAM1
ITGA4 - (ITGB7+VCAM1)
ITGA4 - (ITGB1+VCAM1)
ICAM1 - ITGAL
F11R - JAM3
F11R - JAM2
CEACAM1 - CEACAM5
CD99 - CD99
CD96 - PVR
SELL - PODXL
OCLN - OCLN
JAM2 - F11R
ITGB2 - ICAM1
ICAM1 - SPN
ICAM1 - ITGAL
ICAM1 - (ITGAX+ITGB2)
ICAM1 - (ITGAL+ITGB2)
HLA-G - LILRB1
HLA-F - LILRB1
F11R - JAM2
ENTPD1 - ADORA2A
CLDN11 - CLDN11
CD96 - PVR
CD8A - CEACAM5
CD86 - CD28
CD80 - CD28
CD80 - CD274
CD6 - ALCAM
CD1D - CEACAM5
sample
placl-neg
placl-pos
p-value
0.01 < p < 0.05
p < 0.01
E
F
G
H
I

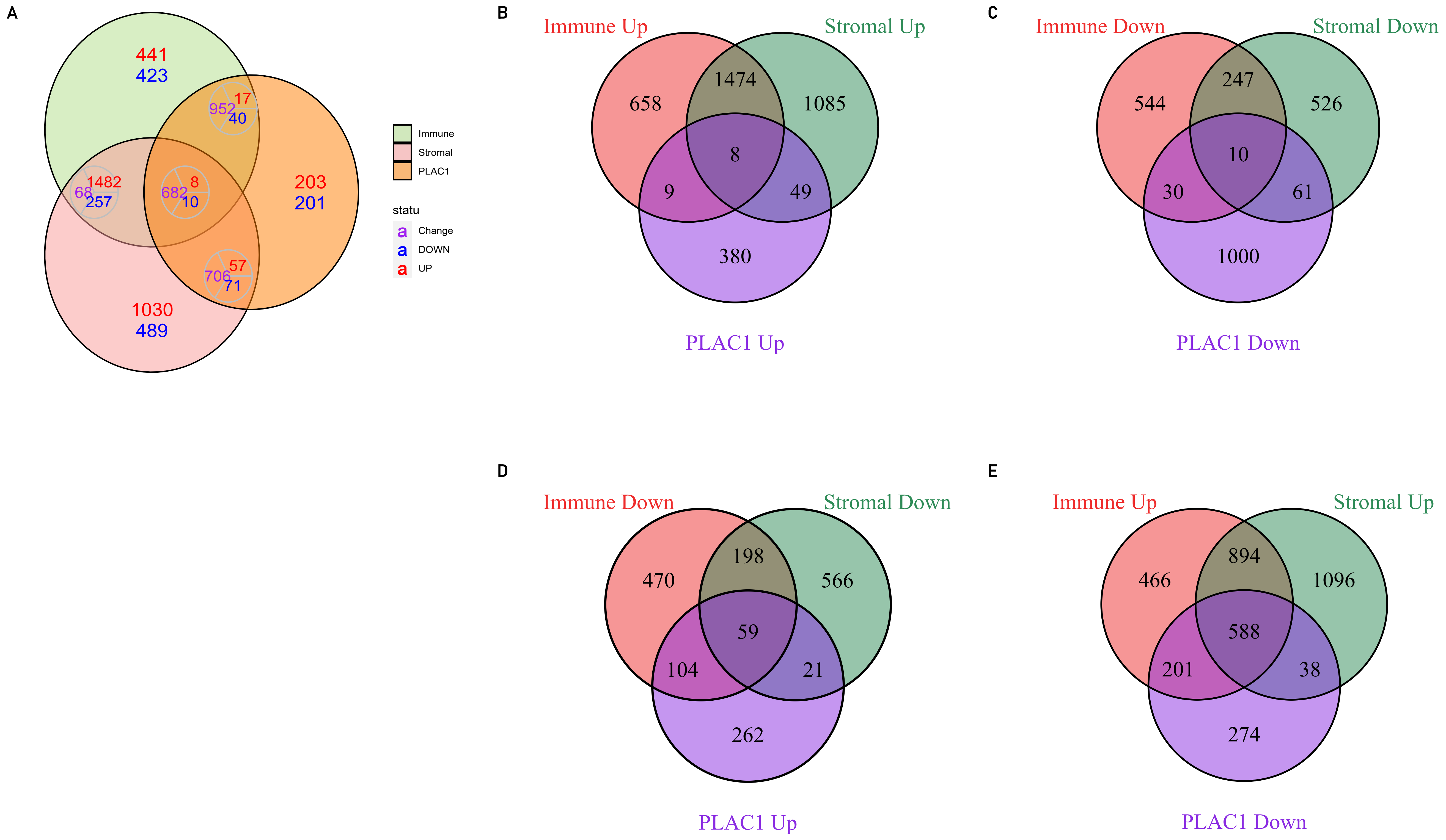

A
441
423
17
952
40
203
201
1482
68
257
8
682
10
57
706
71
1030
489
Immune
Stromal
PLAC1
statu
a Change
a DOWN
a UP
B
Immune Up
Stromal Up
658
1474
1085
8
9
49
380
PLAC1 Up
C
Immune Down
Stromal Down
544
247
526
10
30
61
1000
PLAC1 Down
D
Immune Down
Stromal Down
470
198
566
59
104
21
262
PLAC1 Up
E
Immune Up
Stromal Up
466
894
1096
588
201
38
274
PLAC1 Down

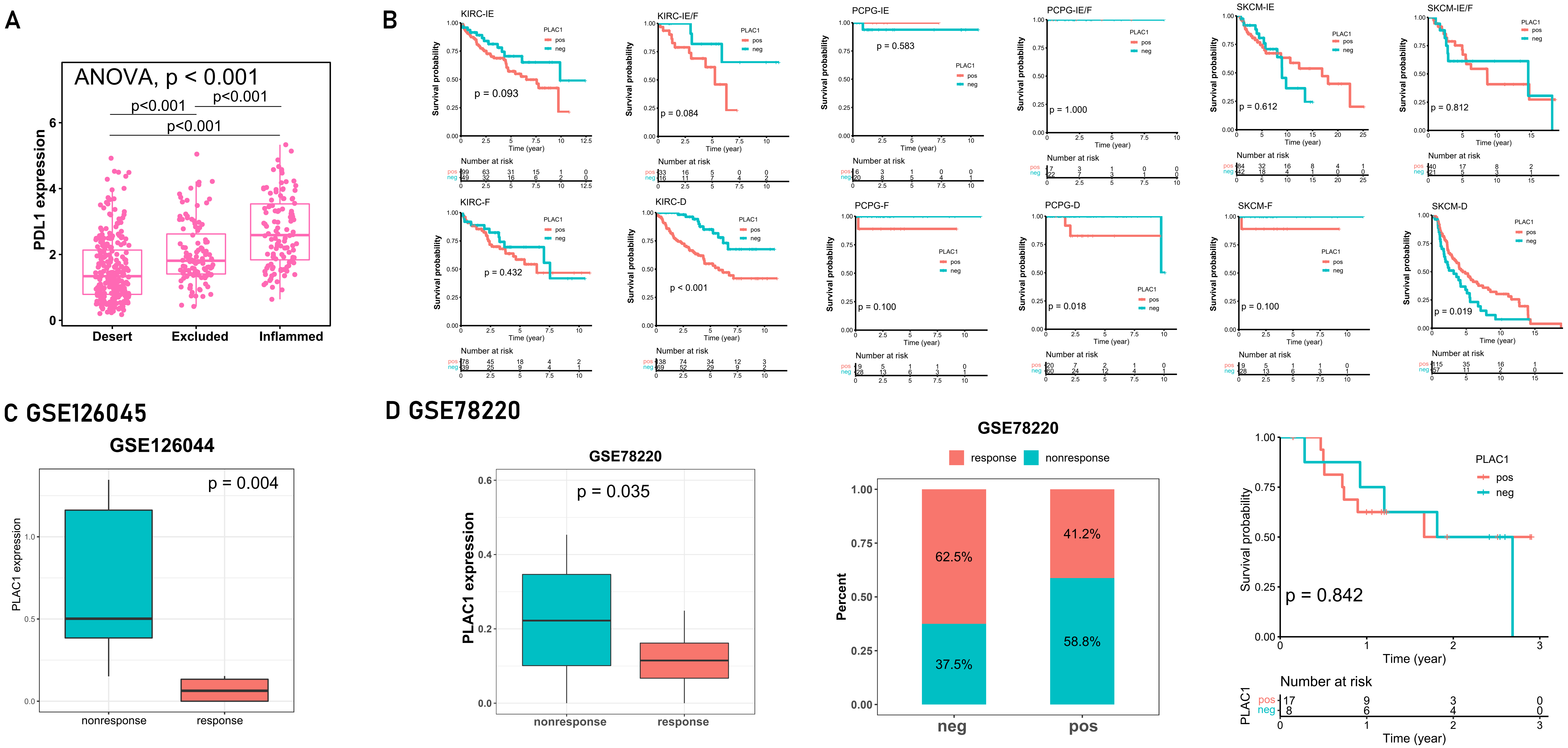
A
ANOVA, p < 0.001
p<0.001
p<0.001
p<0.001
PDL1 expression
Desert
Excluded
Inflammed
B
KIRC-IE
p = 0.093
KIRC-IE/F
p = 0.084
PCPG-IE
p = 0.583
PCPG-IE/F
p = 1.000
SKCM-IE
p = 0.612
SKCM-IE/F
p = 0.812
KIRC-F
p = 0.432
KIRC-D
p < 0.001
PCPG-F
p = 0.100
PCPG-D
p = 0.018
SKCM-F
p = 0.100
SKCM-D
p = 0.019
PLAC1
pos
neg
Survival probability
Time (year)
Number at risk
C GSE126045
GSE126044
p = 0.004
PLAC1 expression
nonresponse
response
D GSE78220
GSE78220
p = 0.035
PLAC1 expression
nonresponse
response
GSE78220
response
nonresponse
Percent
62.5%
37.5%
41.2%
58.8%
neg
pos
PLAC1
pos
neg
Survival probability
p = 0.842
Time (year)
Number at risk
PLAC1
pos 17 9 3 0
neg 8 6 4 0
Time (year)

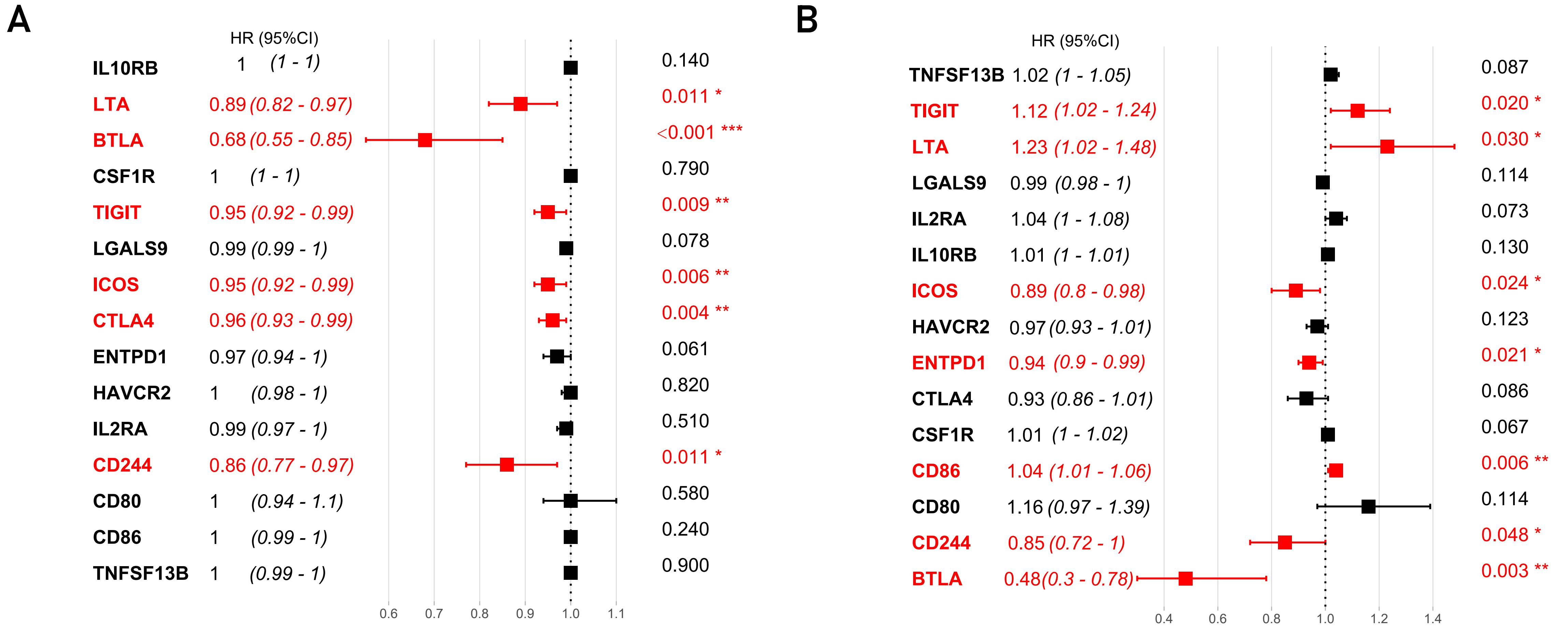

A
HR (95%CI)
IL10RB 1 (1 - 1) 0.140
LTA 0.89 (0.82 - 0.97) 0.011 *
BTLA 0.68 (0.55 - 0.85) <0.001 ***
CSF1R 1 (1 - 1) 0.790
TIGIT 0.95 (0.92 - 0.99) 0.009 **
LGALS9 0.99 (0.99 - 1) 0.078
ICOS 0.95 (0.92 - 0.99) 0.006 **
CTLA4 0.96 (0.93 - 0.99) 0.004 **
ENTPD1 0.97 (0.94 - 1) 0.061
HAVCR2 1 (0.98 - 1) 0.820
IL2RA 0.99 (0.97 - 1) 0.510
CD244 0.86 (0.77 - 0.97) 0.011 *
CD80 1 (0.94 - 1.1) 0.580
CD86 1 (0.99 - 1) 0.240
TNFSF13B 1 (0.99 - 1) 0.900
0.6 0.7 0.8 0.9 1.0 1.1
B
HR (95%CI)
TNFSF13B 1.02 (1 - 1.05) 0.087
TIGIT 1.12 (1.02 - 1.24) 0.020 *
LTA 1.23 (1.02 - 1.48) 0.030 *
LGALS9 0.99 (0.98 - 1) 0.114
IL2RA 1.04 (1 - 1.08) 0.073
IL10RB 1.01 (1 - 1.01) 0.130
ICOS 0.89 (0.8 - 0.98) 0.024 *
HAVCR2 0.97 (0.93 - 1.01) 0.123
ENTPD1 0.94 (0.9 - 0.99) 0.021 *
CTLA4 0.93 (0.86 - 1.01) 0.086
CSF1R 1.01 (1 - 1.02) 0.067
CD86 1.04 (1.01 - 1.06) 0.006 **
CD80 1.16 (0.97 - 1.39) 0.114
CD244 0.85 (0.72 - 1) 0.048 *
BTLA 0.48 (0.3 - 0.78) 0.003 **
0.4 0.6 0.8 1.0 1.2 1.4

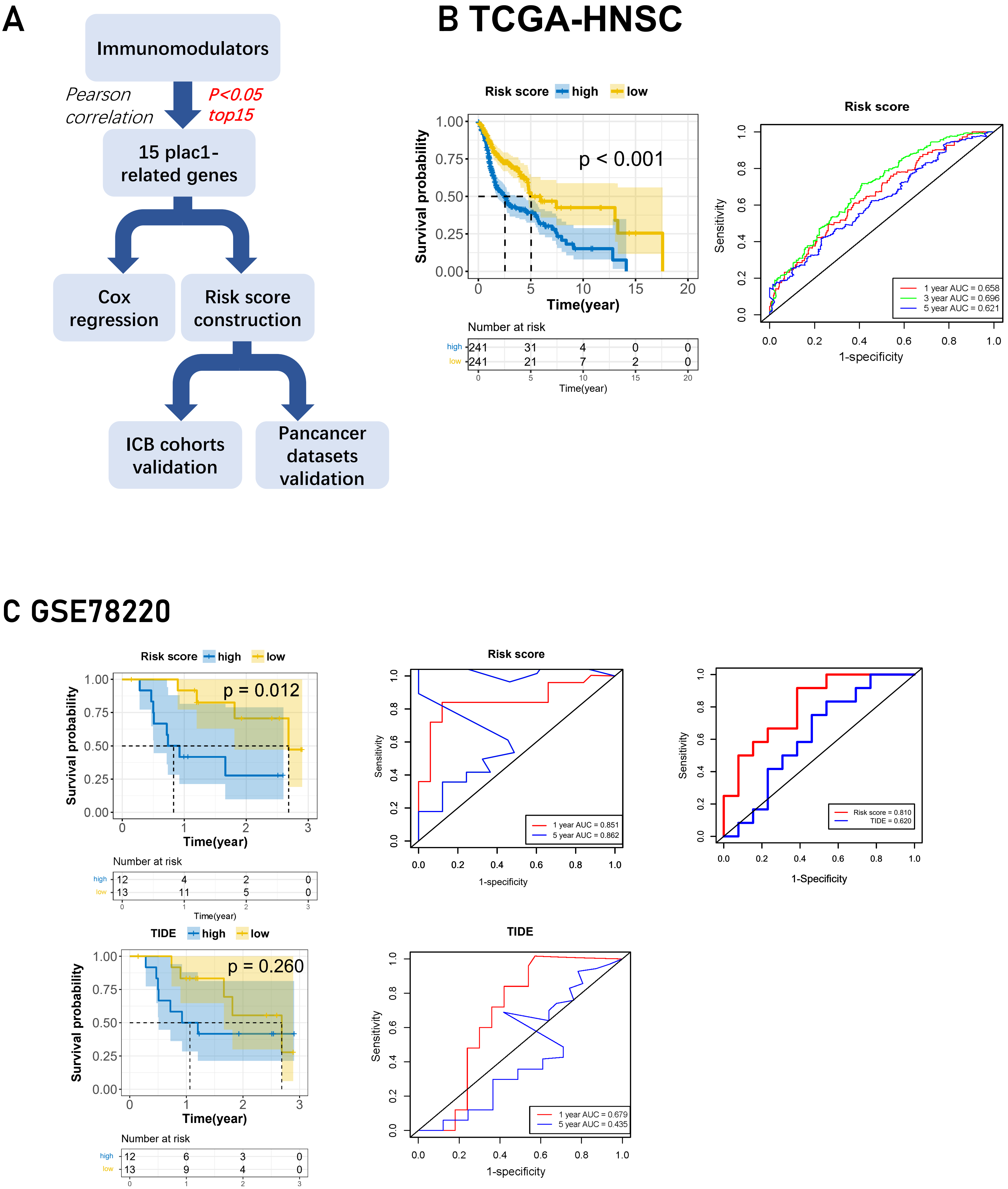
A
Immunomodulators
Pearson correlation
P<0.05
top15
15 plac1-related genes
Cox regression
Risk score construction
ICB cohorts validation
Pancancer datasets validation
B TCGA-HNSC
Risk score high low
p < 0.001
Survival probability
Time(year)
Number at risk
high 241 31 4 0 0
low 241 21 7 2 0
Risk score
Sensitivity
1-specificity
1 year AUC = 0.658
3 year AUC = 0.696
5 year AUC = 0.621
C GSE78220
Risk score high low
p = 0.012
Survival probability
Time(year)
Number at risk
high 12 4 2 0
low 13 11 5 0
Risk score
Sensitivity
1-specificity
1 year AUC = 0.851
5 year AUC = 0.862
Sensitivity
1-Specificity
Risk score = 0.810
TIDE = 0.620
TIDE high low
p = 0.260
Survival probability
Time(year)
Number at risk
high 12 6 3 0
low 13 9 4 0
TIDE
Sensitivity
1-specificity
1 year AUC = 0.679
5 year AUC = 0.435

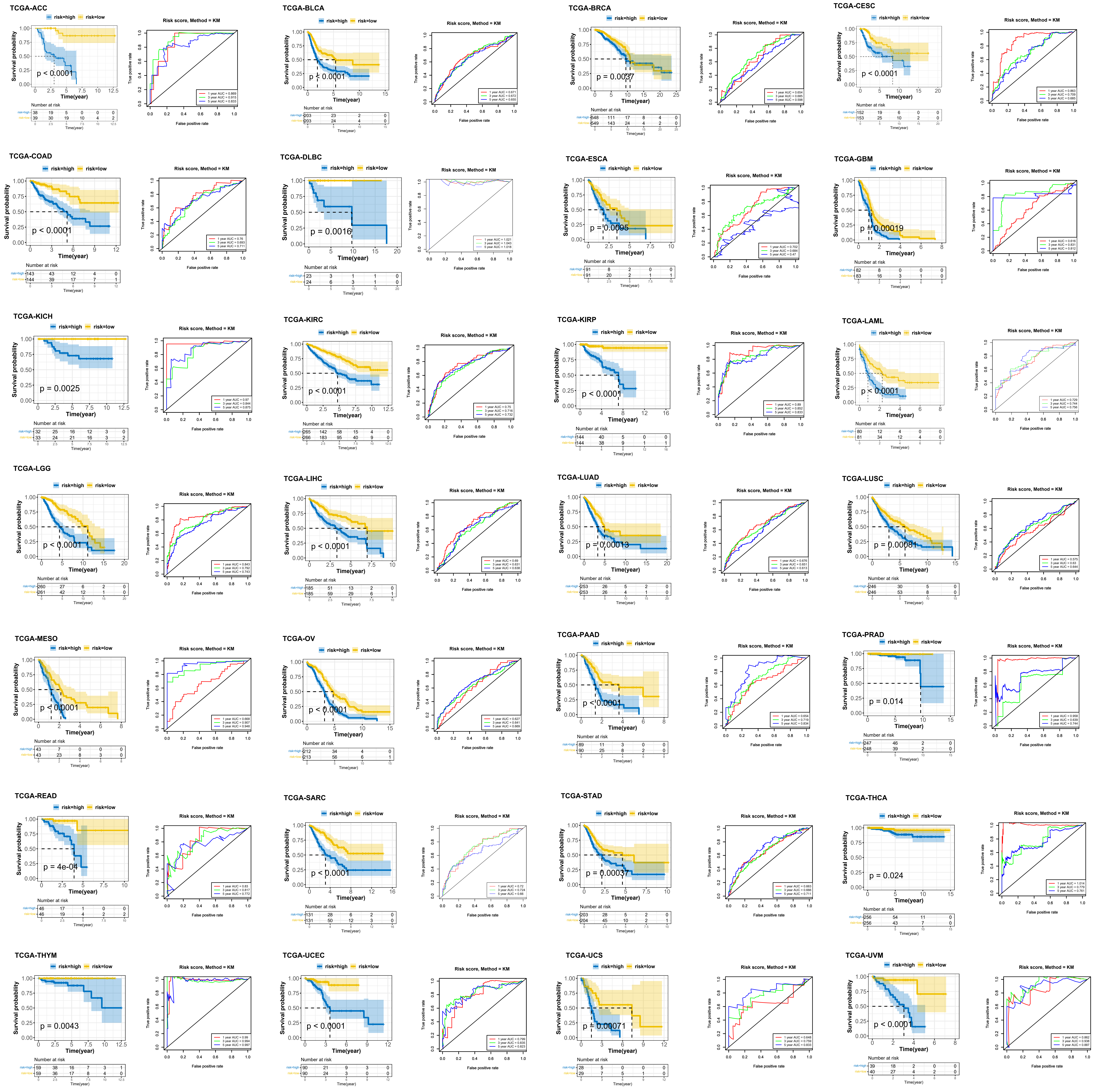

TCGA-ACC
p < 0.0001
TCGA-BLCA
p < 0.0001
TCGA-BRCA
p = 0.0037
TCGA-CESC
p < 0.0001
TCGA-COAD
p < 0.0001
TCGA-DLBC
p = 0.0016
TCGA-ESCA
p = 0.0095
TCGA-GBM
p = 0.00019
TCGA-KICH
p = 0.0025
TCGA-KIRC
p < 0.0001
TCGA-KIRP
p < 0.0001
TCGA-LAML
p < 0.0001
TCGA-LGG
p < 0.0001
TCGA-LIHC
p < 0.0001
TCGA-LUAD
p = 0.00013
TCGA-LUSC
p = 0.00081
TCGA-MESO
p < 0.0001
TCGA-OV
p < 0.0001
TCGA-PAAD
p < 0.0001
TCGA-PRAD
p = 0.014
TCGA-READ
p = 4e-04
TCGA-SARC
p < 0.0001
TCGA-STAD
p = 0.00037
TCGA-THCA
p = 0.024
TCGA-THYM
p = 0.0043
TCGA-UCEC
p < 0.0001
TCGA-UCS
p = 0.00071
TCGA-UVM
p < 0.0001
risk=high
risk=low
Survival probability
Time(year)
Number at risk
Risk score, Method = KM
True positive rate
False positive rate

Supplementary Figure legends

Figure S1 Overview of the study design.

First, we searched public databases. Next, we delineated the expression pattern, prognostic significance and biological characteristics of plac1. Then, we demonstrated the strong correlation between plac1 and the noninflamed TME using bulk and single-cell data. Finally, we constructed a 15-gene signature to represent patient risk in immunotherapy and validated the signature in multiple ICB cohorts.

Figure S2

(A) The expression status of plac1 in multiple cancers was analyzed based on data from TCGA. BLCA: Bladder Urothelial Carcinoma; BRCA: Breast invasive carcinoma; CHOL: Cholangiocarcinoma; COAD: Colon adenocarcinoma; ESCA: Esophageal carcinoma; KIRC: Kidney renal clear cell carcinoma; LIHC: Liver hepatocellular carcinoma; LUAD: Lung adenocarcinoma; LUSC: Lung squamous cell carcinoma; PAAD: Pancreatic adenocarcinoma; READ: Rectum adenocarcinoma; STAD: Stomach adenocarcinoma; THCA: Thyroid carcinoma; UCEC: Uterine Corpus Endometrial Carcinoma

(B) The expression status of plac1 in various cancer single-cell datasets was analyzed through TISCH.

(C) The expression status of plac1 in all cell types in the single-cell dataset GSE103322.

(D-E) Differences in plac1 expression in different phases during tumorigenesis and metastasis based on data from GSE30784 (D) and GSE78060 (E).

Figure S3

(A) Gene alteration features of plac1 in plac1-positive and plac1-negative samples.

(B-C) Gene alteration profiles of driver genes in plac1-positive (B) and plac1-negative (C) samples.

(D) Differences in driver gene expression levels in plac1-positive and plac1-negative samples.

*p<0.05, **p<0.01, ***p<0.001

Figure S4

(A-B) Correlation between plac1 expression and disease-free survival (DFS) (A) and metastasis-free survival (MFS) (B).

(C-F) Correlation between plac1 expression and overall survival (OS) in early-stage patients (C), non-lymph node metastasis patients (D), female patients (E), hypopharynx cancer patients (F), and oral cavity cancer patients (G), respectively.

(H) Expression level of plac1 in samples with different HPV statuses from the TCGA-HNSC dataset.

(I) Sample counts of plac1-negative and plac1-positive cells with different HPV statuses from the TCGA-HNSC dataset.

(J) Correlation between HPV status and patient survival in plac1-positive samples from the TCGA-HNSC dataset.

(K) Immune score in HNSC of different sites.

Figure S5

(A) Correlations between plac1 and the enrichment scores of anti-EGFR therapy signature.

(B) Correlations between plac1 and the enrichment scores of several therapeutic signatures.

(C) Differences in EGFR gene expression levels between the plac1-positive and plac1-negative groups.
(D) Expression levels of plac1 in the anti-EGFR therapy nonresponse and response groups.
*p<0.05, **p<0.01, ***p<0.001

Figure S6
(A) GSEA of metabolism in DEGs between plac1-positive and plac1-negative samples.
(B) GSEA of KEGG pathway gene sets in plac1-positive and plac1-negative cells from the single-cell dataset GSE103322.
*p<0.05, **p<0.01, ***p<0.001

Figure S7
(A-B) Differences in ILC1 (A) and ILC3 (B) marker gene expression in plac1-positive and plac1-negative samples.

Figure S8
(A) GSEA of immune-related hallmarks in DEGs between plac1-positive and plac1-negative samples.
(B) Plac1 was negatively correlated with the immune score.
(C) Correlations between plac1 and immune infiltration level.
(D) Correlation between plac1 and the infiltration levels of six types of immune cells (T cells, $CD4^+$ T cells, $CD8^+$ T cells, cytotoxic lymphocytes, dendritic cells, and B cells), which were calculated using five independent algorithms.
(E-G) Differences in the enrichment score or expression level of immunomodulators (E), immune effector genes (F), and immune checkpoints (G) in plac1-positive and plac1-negative samples.
*p<0.05, **p<0.01, ***p<0.001

Figure S9
(A) Correlations between immune score and patient survival in plac1-positive and plac1-negative samples.
(B) Correlations between plac1 status and patient survival in samples with T cell infiltration level.

Figure S10
(A-C) Differences in cell-cell interactions among all cells in the TME between plac1-positive and plac1-negative samples. (A) represents cell-cell contact, (B) represents ECM-receptor interaction, and (C) represents secreted signaling.
(D-I) Cell-cell interaction analysis of the TME. Data were obtained from the single-cell sequencing dataset GSE103322. (D-F) represent the interaction strength from cell X to cell Y, whereas (G-I) represent the interaction strength from cell Y to cell X. (D and G) represent cell-cell contact, (E and H) represent ECM-receptor interaction, and (F and I) represent secreted signaling.

Figure S11
Differentially expressed RNAs between the plac1 groups, the immune score groups and the stromal

score groups.
(A) The total number of differentially expressed genes in the three groups.
(B) There were 17 and 57 common RNAs between RNAs upregulated among the plac1-positive group and RNAs upregulated among the high immune score groups and high stromal groups, respectively.
(C) There were 40 and 71 common RNAs between RNAs downregulated among the plac1-positive group and RNAs downregulated among the high immune score groups and high stromal groups, respectively.
(D) There were 163 and 80 common RNAs between RNAs upregulated among the plac1-positive group and RNAs downregulated among the high immune score groups and high stromal groups, respectively.
(E) There were 789 and 626 common RNAs between RNAs downregulated among the plac1-positive group and RNAs upregulated among the high immune score groups and high stromal groups, respectively.

Figure S12
(A) The differences of PD-L1 expression levels in three immune phenotypes validated the rationality of this classification. One-way ANOVA test $p<0.001$; dessert vs excluded, dessert vs inflamed, excluded vs inflamed: $p<0.001$.
(B) The correlations between plac1 status and patient survival in four TME subtypes in KIRC, PCPG, and SKCM.
(C) Differences in plac1 expression in the response and nonresponse groups in the immunotherapy cohort. Data are obtained from GSE126045.
(D) Correlation between plac1 and clinical response to immunotherapy. Data are obtained from GSE78220.

Figure S13
(A-B) Univariate (A) and multivariate (B) Cox regression analysis of TCGA-HNSC for genes in 15-gene signature.
*$p<0.05$, **$p<0.01$, ***$p<0.001$

Figure S14
(A) Flow chart of developing a plac1-related risk score.
(B) Validation of the prognostic value of the risk score in the TCGA-HNSC dataset.
(C) Validation of the prognostic value of the risk score in the GSE78220 dataset compared with the TIDE algorithm.

Figure S15
Validation of the prognostic value of the risk score across cancers. The correlation between risk score and patient survival in different cancer types is shown.
